# Mimicking nature to develop halide perovskite semiconductors from proteins and metal carbonates

**DOI:** 10.1038/s41598-024-66116-8

**Published:** 2024-07-04

**Authors:** Masoud Aminzare, Yangshixing Li, Sara Mahshid, Noémie-Manuelle Dorval Courchesne

**Affiliations:** 1 Department of Chemical Engineering, McGill University, Montreal, Canada; 2 Department of Bioengineering, McGill University, Montreal, Canada

**Keywords:** Halide perovskites, Metal carbonates, Proteins, Bio-templates, Biomineralization, Ion exchange, Biosynthesis, Chemical engineering, Inorganic chemistry, Materials chemistry, Chemical synthesis, Chemical engineering, Materials science, Materials for devices, Nanoscale materials

## Abstract

Halide perovskite (HPs) nanostructures have recently gained extensive worldwide attentions because of their remarkable optoelectronic properties and fast developments. However, intrinsic instability against environmental factors—i.e., temperature, humidity, illumination, and oxygen—restricted their real-life applications. HPs are typically synthesized as colloids by employing organic solvents and ligands. Consequently, the precise control and tuning of complex 3D perovskite morphologies are challenging and have hardly been achieved by conventional fabrication methods. Here, we combine the benefits of self-assembly of biomolecules and an ion exchange reaction (IER) approach to customize HPs spatial shapes and composition. Initially, we apply a biomineralization approach, using biological templates (such as biopolymers, proteins, or protein assemblies), modulating the morphology of MCO_3_ (M = Ca^2+^, Ba^2+^) nano/microstructures. We then show that the morphology of the materials can be maintained throughout an IER process to form surface HPs with a wide variety of morphologies. The fabricated core–shell structures of metal carbonates and HPs introduce nano/microcomposites that can be sculpted into a wide diversity of 3D architectures suitable for various potential applications such as sensors, detectors, catalysis, etc. As a prototype, we fabricate disposable humidity sensors with an 11–95% detection range by casting the formed bio-templated nano/micro-composites on paper substrate.

## Introduction

Materials with complex 3D morphologies and promising properties are eminently desirable for many applications, including catalysts, sensors, and optoelectronic devices. Correspondingly, accurate control over morphology, composition, and uniformity is required during synthesis. Various bottom-up and top-down approaches have been employed to construct such 3D arrangements. However, either shape or composition can be accurately tuned using existing self-assembly approaches. In other words, obtaining both favourable spatial structure and preferred chemical composition simultaneously at the nano/microscale is challenging for many valuable material systems while relatively accessible for others. This is because several parameters must be precisely tuned, including chemical reaction equilibria, reaction thermodynamics and kinetics, crystal nucleation and growth, surface functionalizing ligands^1–4^.

For example, metal carbonates (MCO_3_; M = Ca^2+^, Ba^2+^, Sr^2+^, etc.) are a family of naturally occurring biominerals that can be manufactured in a variety of crystalline shapes and morphologies by means of environmentally friendly biological and bio-inspired mineralization routes^5–14^. However, the resulting carbonate hierarchical materials have restricted functionalities because of their limited intrinsic chemical compositions. On the other hand, many promising functional advanced materials are challenging to assemble into complex shapes. Halide perovskites (HPs), for instance, have emerged as a remarkable new class of materials with outstanding optoelectrical properties over the last decade. They possess promising optoelectrical properties such as suitable and tunable bandgap, balanced carrier mobility, defect tolerability, high photoluminescence quantum yields, etc., which lead to their rapid and remarkable development^15^. Although their versatility of synthesis and relatively simple scale-up are promising for many usages, spatially assembled HPs nano/microstructures are critically required for integrated advanced applications^1,16^. HP nanoparticles are routinely synthesized in organic solvents and with organic ligands (most of them are harmful to the environment). The caveat, though, is that complex 3D nano/microstructures of HPs are difficult to organize by conventional synthesis routes, as the crystallization of HPs is a remarkably rapid phenomenon, making it challenging to explore and monitor their growth pathway. Likewise, 3D patterning of HP thin films generally requires advanced techniques with specific instruments that are complicated, non-efficient, sluggish, arduous, and challenging to scale up^1,17–27^.

In solar energy conversion applications, it is well known that the optoelectronic properties of HPs are strongly contingent on their structural and morphological dimensionality^28^. Hence, developing novel approaches for rapid, straightforward, and scalable construction of spatial assemblies of HP individual nano/microparticles and thin films is highly in demand.

To circumvent the abovementioned challenges and obtain nano/micromaterials with specific composition and desirable features (e.g., size and shape), post-synthetic modifications of complex preformed 3D structures can be applied. For a long time now, researchers and scientists have smartly employed chemical reaction principles to convert naturally available and widely abundant materials into more valuable and functional compounds for advanced applications while preserving the valuable properties of the primary material^29^. For instance, metals and metal oxides have already been synthesized using oxidation/reduction reactions by converting both biological and synthetic materials^30–32^. Ion exchange is another powerful and versatile approach to tune the material composition and obtain many precious compounds, such as semiconductors, which can hardly be fabricated synthetically using conventional routes. Thereby, ionic micro/nanostructures can be converted into a broad variety of compositions with the advantage of preserving the original morphology^3,33–38^. There are two types of ion exchange reactions (IERs): cation exchange and anion exchange, with the latter being more difficult due to the larger size and lower diffusivity of anions^3,34,36,39^. Technically, the chemical transformation of present 3D arrangements into a particular composition provides control over both the chemistry and spatial geometry. Succinctly in the IER processes, the cation or anion sublattice of a pre-programmed nano/microstructure is substituted with different ions to manufacture superior material that possess exceptional morphology and homogeneity. The IER procedure is applicable for either partial compositional conversions through either cation or anion substitutions or full transformations by combining sequential cation–anion IERs^1,3,5,39,40^.

Effective and independent control over structure and composition during IER conversion depends on many factors, including but not limited to (1) the existence of fine nanocrystals (preferably 5–50 nm) to provide improved chemical reactivity, (2) the accommodation of volume changes during transformation by material scaffold, (3) the minimization of dissolution/recrystallization reactions to hinder/terminate formation of new nuclei and nanocrystals growth to avoid morphological alterations, (4) atomistic unit cell conformity with minimal rearrangements of the crystal lattice, (5) proper solid-state diffusion of reactants, (6) fast transport of ions toward and away from the nanoparticles within the whole assembly as well as precise control over (7) reagents concentrations, (8) pH (in case of solution conversion), (9) temperature^1,3,5,40^.

There are several examples of applying IER methods to obtain advanced materials with preserved structures^41^. Hendrikse et al. has recently studied the transformation of barium carbonates (BaCO_3_) from coral-, spiral-, vase-, and post-like shaped BaCO_3_/SiO_2_ composites into cadmium-, iron-, manganese-, and nickel-sulfides and oxides by a two-step IER process^40^. IER process is applied not only for ionic compounds but also to assemble materials with covalent characteristics. Beberwyckc and colleagues employed cation IER to synthesize III–V semiconductors, i.e., GaAs, InAs, GaP, and InP, from II–V nanocrystals, i.e., cadmium pnictides while maintaining original monodispersity and shape^42^. Powell et al. converted roxbyite-type Cu_2−x_S nanocrystals to wurtzite-type CoS and MnS via a cation IER. These are metastable polymorphs (in bulk systems), though they could be formed by IER thanks to the conservation of features of cation and anion sublattices^43^.

Substitution of halide ions in HPs is somewhat simple and occurs at room temperature owing to spontaneous migration of halide ions. It has been applied to the formation of lead HP nanocrystals of different shapes to adjust their emission color. Moreover, cation IER has also been employed to acquire mixed cation HP nanocrystals with distinct optoelectrical features^16,44^. Likewise, it has been demonstrated that the formation of spatially arranged HPs with interesting optoelectronic properties can be facilitated via IER from metal carbonates due to the crystal lattice similarities^29,45^. Holtus et al.^5^ has recently converted pre-programmed metal carbonate microstructures into HPs with the morphological and crystallinity retention of the initial micro-architectures. The process consists of introducing lead into the structure by cation IER, followed by anion exchange of carbonate group with halide moieties, together with rapid methylammonium insertion to produce HPs^5^. From a more recent work from the same group by Helmbrecht et al.^1^, ion exchange lithography was used to spatially pattern MAPbBr_3_ and FAPbBr_3_ semiconductors films by printing ion exchange “inks” on a reactive nanoparticle “canvas”. In this regard, a precisely controlled transformation of an electrically insulating canvas into semiconducting HPs happens through printing, painting, and spraying reactive inks on an ion exchange reactive canvas. Besides, they demonstrated that the ion exchange lithography methodology could be incorporated into the construction of optoelectronic devices such as light-emitting diodes^1^.

Molecular self-assembly using biological macromolecules provides means for bottom-up fabrication methods to nucleate, bind and organize novel nanostructured inorganic materials at low costs and with a wider range of morphologies. Inspired by biomineralization in nature, biomolecules and biomaterials, such as proteins and microorganisms, can provide nucleation sites to bind and organize nanocrystals and be rationally designed to enhance materials affinity and control the growth of nanocrystals^46–50^. These bio-species may serve as ‘natural’ reaction vessels and nanoscale bio-templates for directing the synthesis of materials with novel and complex morphologies. In this regard, various functional protein–inorganic composites with complex and well-defined structures have been synthesized. Proteins as simple as bovine serum albumin (BSA) can direct the growth of inorganic nanocrystals. For instance, protein templates have organized gold nanoparticles, quantum dots, and oxides at the nanoscale^50–52^. Copper phosphate nanoflowers were synthesized via ion nucleation by BSA, leading to the formation of branched petal-like shapes. In this case, simple electrostatic interactions contribute to nucleating ions, the protein acting as a biological ligand^53^. Likewise, fibrous proteins, such as collagen, chitin, filamentous phages, and amyloid fibers, represent bio-scaffolds of interest to synthesize nanowire-shaped objects^54,55^.

To the best of our knowledge, there have been rare reports of designing biomolecules-HP complexes so far^56–58^. Therefore, inspired by previous studies and nature, we aim to implement biomaterials (proteins, viruses) as nucleation templates to produce metal carbonates with desirable morphologies followed by subsequent IER to form HP nano/microstructures while preserving the original shape obtained by biomineralization. Using this methodology, we target towards (i) controlling the structure of HPs at the nano- and microscale, (ii) greener synthesis of HP nanostructures, and (iii) producing optically active nano/microcomposites. Our fabrication strategy provides a systematic, versatile, and customizable approach towards a wide range of HP morphologies with independent control over the chemical compositions, i.e., applying different halide ions. Also, we determine the effect of reaction time on conversion and structural evolutions during both IER steps, which has not been investigated before for HPs^5^. Finally, we showcased the capability of our bioinspired HPs for practical environmentally friendly usages by applying synthesized HP nano/micro-structures for humidity sensing applications. This method enables the conversion of bioinspired materials into semiconductors, equipping biological and programmable synthetic morphologies with the performance of artificial perovskite-based semiconductors.

## Results and discussion

### Biomineralization of metal carbonates

**Figure 1:**
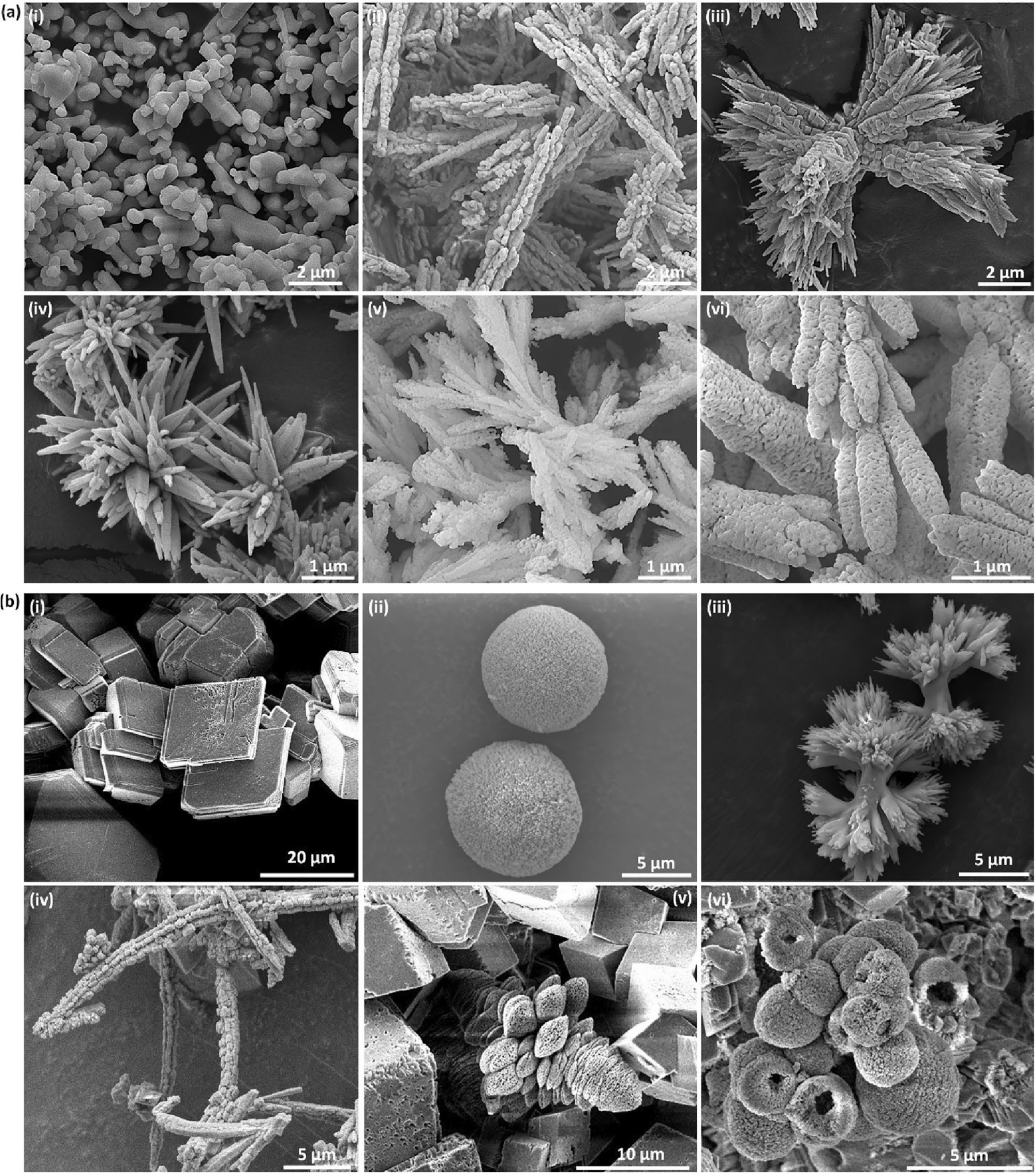
Diversity of carbonate nano/microstructures formed with bio-templates. (**a**) Various BaCO_3_ nano/microstructures synthesized with (i) no bio-template, (ii) starch, (iii, iv) M13 bacteriophage, and (v, vi) BSA. (**b**) Different CaCO_3_ nano/microstructures formed with (i) no template, (ii, iii) starch, and (iv–vi) dispersed curli fibers.

**Figure 2:**
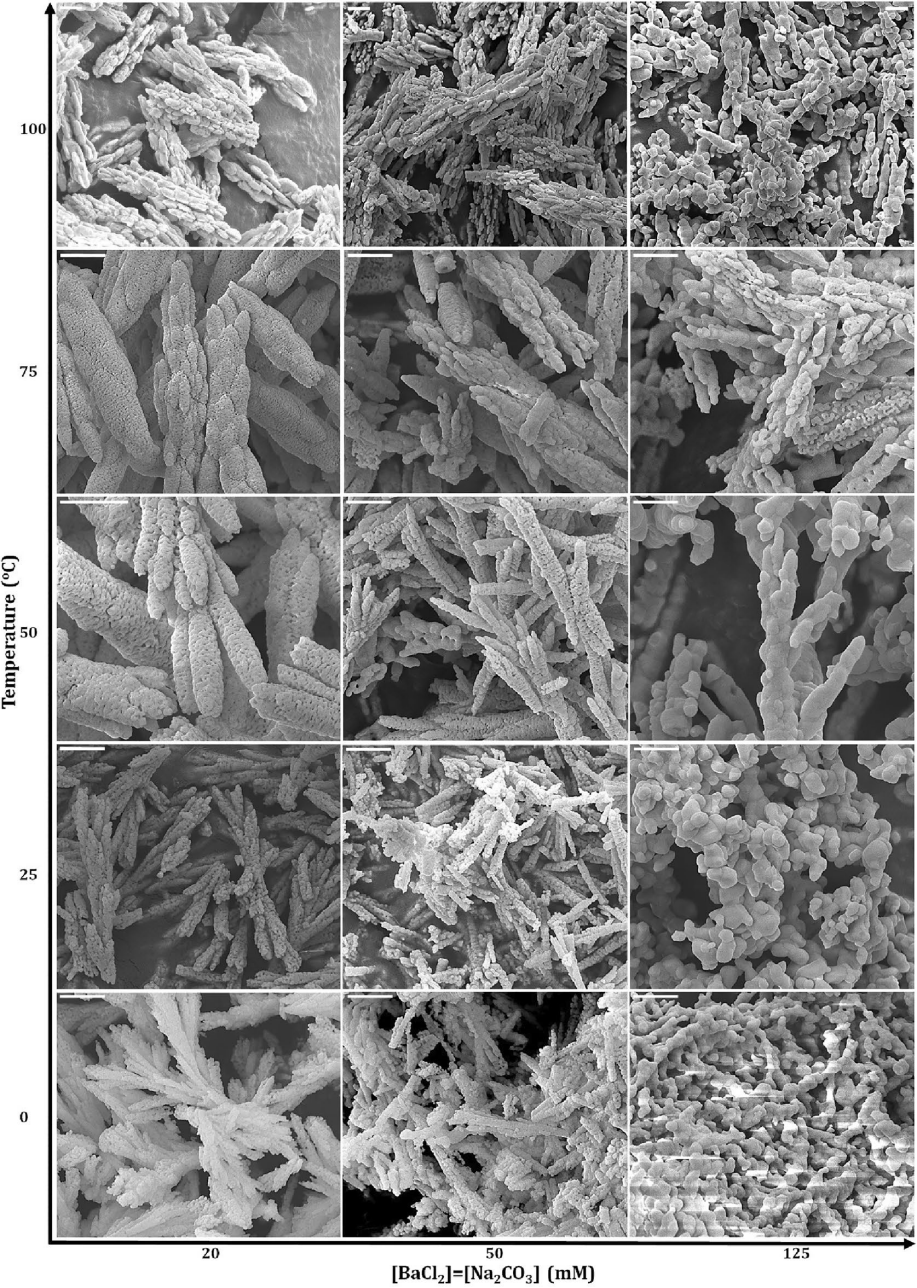
BaCO_3_ nano/microstructures with different morphologies synthesized with various solution temperature and precursors concentration using BSA as bio-template (scale bars: 1 µm).

Biomineralization, as a nature-inspired approach in the sense of employing biological or bioinspired compounds as templates or growth modifiers, provides possibilities to produce nano/microscale crystals with incredibly diverse morphologies. Here we used biomineralization to form metal carbonates with various morphologies that we then converted to HPs while maintaining the original shape. Figure 1a shows the morphological variety of BaCO_3_ nano/microstructures synthesized by using bio-template method. While the shape of synthesized nano/microstructures can be hardly changed by merely altering synthesis parameters such as temperature and precursors concentrations (Fig. S1), employing bio-templates provides simple but powerful tools to produce complex nano/microstructures like stars, dumbbells, elongated morphologies, hierarchical, porous rods and branched mesoporous shapes (Fig. 2 and S2). In addition, bio-templates and synthesis parameters can be used simultaneously to control the complexity of the morphologies better and acquire desirable shapes. Besides BaCO_3_, we also synthesized calcium carbonate (CaCO_3_) nano/microstructures (Fig. 1b). CaCO_3_, as an abundant metal carbonate found in nature, has various polymorphs ranging from amorphous (amorphous CaCO_3_ (ACC)) to crystalline and from anhydrous (calcite, aragonite, and vaterite) to differently hydrated (calcium carbonate monohydrate and calcium carbonate hexahydrate)^59–61^. Calcite crystallizes in the hexagonal crystal system, forming face-centered rhombohedral unit cells^62^. On the other hand, aragonite occurs in the orthorhombic system but shares similar crystal structures with calcite in the sense of having alternating layers of carbonate and calcium ions perpendicular to the c axis. These structural similarities between aragonite and calcite lead to their similar thermodynamic stabilities. However, other distinct properties of the two phases result from the elevation of some carbonate ions in the c direction observed in aragonite, which gives rise to the formation of two layers of carbonate ions with different orientations^61^. Vaterite, generally as an intermediate polymorph during the transition from ACC to calcite, crystallizes in the hexagonal system with carbonate ions oriented parallel to the c axis. It is a loosely packed but much more complicated crystalline structure^59,61^. Here, CaCO_3_ 3D assemblies are prepared with the same procedure using soluble starch and dispersed curli fibers^54,63^ as bio-templates (Fig. 1b). Similarly, the bio-templates can significantly alter the morphology of CaCO_3_ samples from cubic shapes (no template used) into spherical porous sponges, hierarchical, elongated rods, irregular particles, flowers, and hollow porous spheres. Temperature and precursor concentrations can be tuned together using bio-templates to better control the obtained morphologies and acquire broad types of nano/microstructures (Fig. S3-4). It is worth mentioning that we selected the current biomolecules to provide diverse types, different template structures, and comprehensive functionalities. Starch and BSA are cheap, abundant, and widely used biopolymers and proteins, respectively. The M13 bacteriophages are filamentous structures with a high aspect ratio (880 nm by 6.5 nm) of interest to produce elongated or branched carbonate shapes^52,64^. Curli fibers are fibrous proteins that can form spider web-like arrangements, hydrogels, and film, with the ability to be genetically engineered to obtain distinct functionalities^54,63,65^.

The formation mechanism of various metal carbonate morphologies has been extensively studied before, so we did not intend to investigate it here. Briefly, the final structures and morphologies of MCO_3_ largely depend on the synthesis technique employed as well as the controlling factors such as pH, temperature, reaction time, solvent ratio, M^2+^ :CO_3_^2-^ ratio, mixing mode, substrate surface roughness, and the presence of additives as crystal growth templates (i.e., additive type and concentration), which have direct impacts on the crystal nucleation and growing process (Fig. S5-9). For example, the surface roughness of the chosen templates is important, as it affects the heterogeneous nucleation process by influencing the atom adhesion onto the substrate, the interfacial energy, and the surface energy. The impacts of additives and pH are correlated; namely, the addition of additives would result in a pH variation in the solution, which further improves the function of the additives, giving rise to the formation of various morphologies. The concentration ratio of M^2+^ and CO_3_^2−^ changes the electrostatic attractions between the positively and negatively charged groups in the solution while controlling the nucleation rate. The reaction temperature also has huge impacts on the polymorph, size, and morphology of MCO_3_ particles. More specifically, the energy of the reactive environment rises with an increasing temperature, which facilitates the formation of high surface energy aragonite (in case of calcium carbonate) crystals with a typical elongated rod-/needle-like shape^6,8,61,62,66–71^. Overall, in this work, the temperature increase is beneficial for particle growth and coarsening and pore size increase (Fig. S5-9), while the number of branches and branches’ diameter decrease by raising the synthesis temperature (Fig. S7). Also, very high temperatures (≈ 100 °C) cause the rough surface of particles and damaged morphologies. In terms of precursors concentration, the higher the precursors ratio, the larger the particles and pores size, as well as the more course the structures. While the number of nucleation sites is constant (i.e., template concentration), a higher concentration of precursors leads to faster grain growth and bigger particles (Fig. S5-9). All these controlling parameters mentioned above have intertwined effects on the formation of various morphologies; hence, they must be carefully controlled during the synthetic process to obtain the desired structures.

### IER conversion to HPs

**Figure 3:**
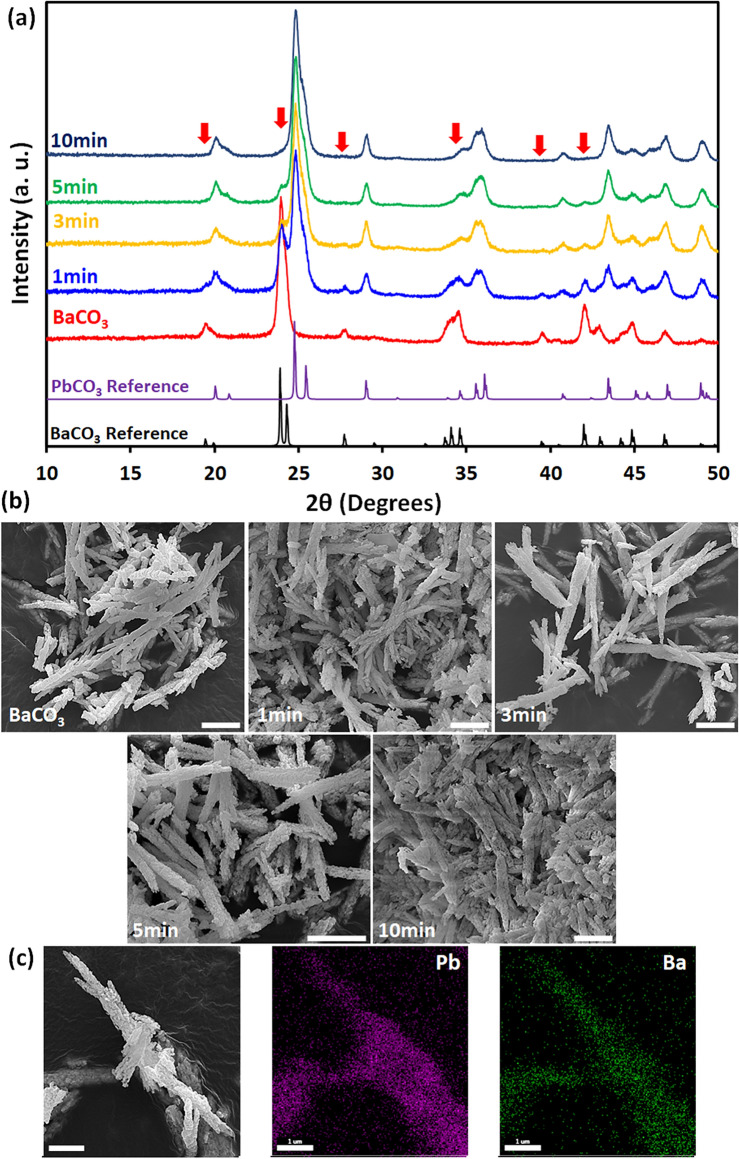
BSA-templated BaCO_3_ nano/microstructures converted to PbCO_3_. (**a**) XRD spectra for different conversion reaction time (1–10 min). Arrows show BaCO_3_ peak positions that are vanishing over IER process. (**b**) SEM micrographs of BaCO_3_ and converted PbCO_3_ with preserved original nano/microstructures (scale bars = 2 µm). (**c**) EDX elemental mapping of converted PbCO_3_ nano/microstructure after 3 min of conversion reaction time (scale bars = 1 µm).

The conversion of metal carbonates into halide perovskite needs two IER steps which must be performed cautiously to preserve the original morphologies. We first started by converting barium carbonate (BaCO_3_) nano/microstructures, as it only crystallizes in the orthorhombic system at ambient temperature^51,52^. For the first IER, Pb^2+^ substitutes M^2+^ in MCO_3_ because PbCO_3_ is thermodynamically more stable (lower solubility in water than other MCO_3_)^5^. However, the dissolution of MCO_3_ and the subsequent nucleation and growth must be avoided to maintain the initial structure during IER. This means the conversion of MCO_3_ into PbCO_3_ has to be completed as fast as possible to hinder nucleation and growth of new PbCO_3_ particles by using high concentrated Pb(NO_3_)_2_ solution (near saturation) to force the cation exchange reaction towards the PbCO_3_ production^5^. Figure 3a represents XRD results of conversion of BaCO_3_ into PbCO_3_ over IER time from 1 to 10 min. Interestingly, strong PbCO_3_ peaks appear even after a very short period, which is an indication of the fast kinetic of cation IER. As time passes, progressively more BaCO_3_ are converted into PbCO_3_, and characteristic XRD peaks of PbCO_3_ become stronger. Eventually, after 10 min, the majority of BaCO_3_ is converted into PbCO_3_, and its XRD representative peaks are almost vanished. SEM micrographs (Fig. 3b) illustrate microstructure evolution of the formation of PbCO_3_ through cation IER. All converted PbCO_3_ samples demonstrate almost identical morphologies to the original BaCO_3_, proving a successful conversion reaction with well-maintained original structures. Furthermore, EDX elemental mapping (Fig. 3c) displays that Pb is homogeneously distributed all over the surface of the sample, meaning that the conversion reaction was unselective and uniform. Ba^2+^ ions are also present in the EDX elemental mapping. This is due to the formation of a core–shell structure, with BaCO_3_ in core and PbCO_3_ on the surface of the nano/microstructures.

**Figure 4:**
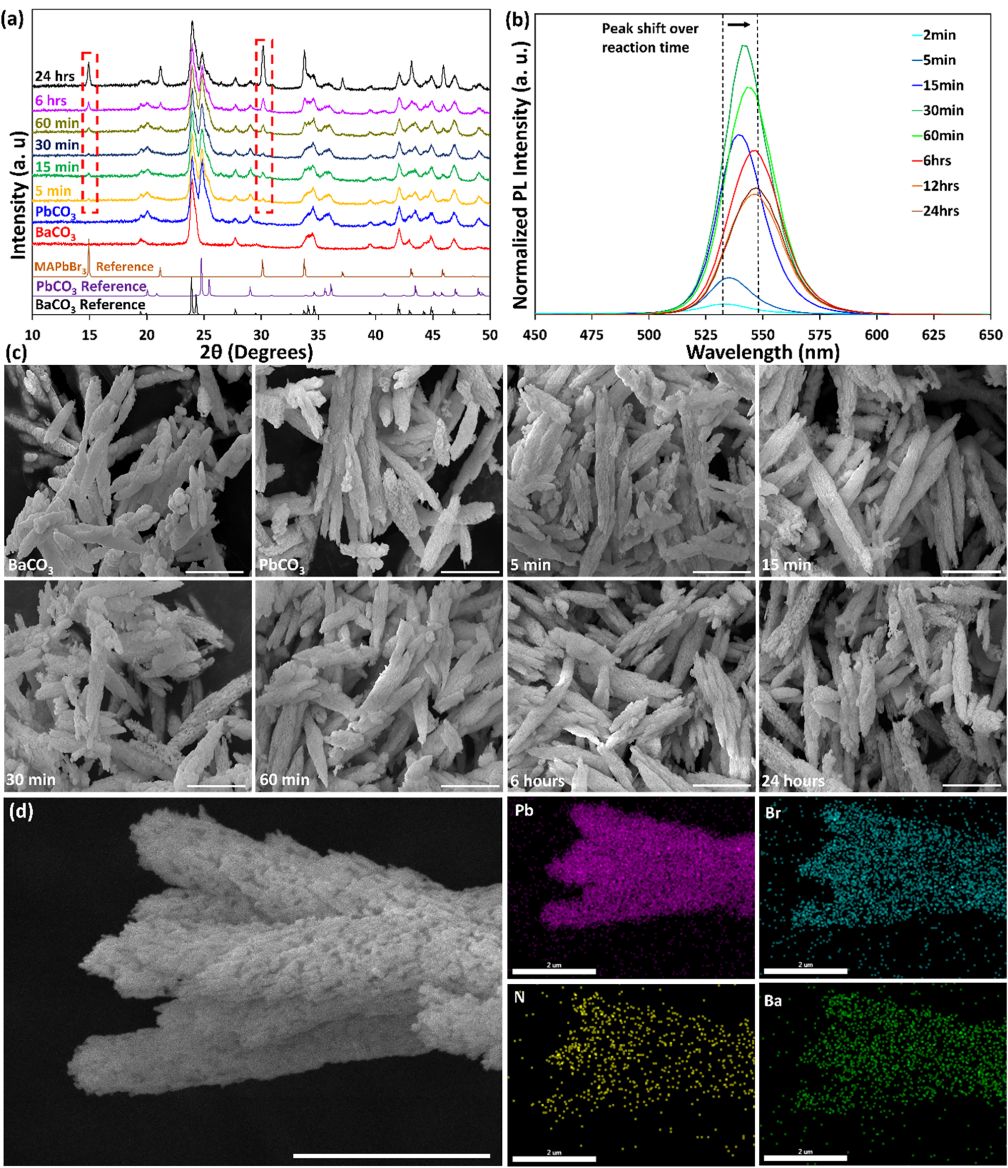
Conversion of BSA-templated BaCO_3_ nano-/microstructures into MAPbBr_3_. (**a**) XRD spectra for different conversion reaction time from 5 min to 24 h. (**b**) PL spectra of converted MAPbBr_3_ nano/microstructures at different conversion reaction time from 5 min to 24 h. (**c**) SEM micrographs of BaCO_3_, PbCO_3_ and converted MAPbBr_3_ with well-maintained original morphologies at different IER time (scale bars = 3 µm). (**d**) EDX elemental mapping of converted MAPbBr_3_ after 30 min conversion reaction time (scale bars = 2 µm).

In the final step, the anion IER is responsible for converting PbCO_3_ into MAPbBr_3_. This step also needs to be performed carefully to preserve the original shape of the metal carbonate. However, as anions have a larger size and lower diffusivity, the anion IER is more difficult and critical than cation IER. In fact, anion IER contains an intermediate step of formation of PbX_2_ composition prior to the full conversion into the halide perovskite^5^. Therefore, it requires well-established insertion of CH_3_NH_3_X and rearrangement of the crystal unit cell during anion IER, making it more difficult and slower than the former cation IER. Unlike the previously reported anion IER of PbCO_3_ to HPs in the gaseous form^5^, we fulfilled this step in liquid form using IPA. The liquid phase transformation can accelerate the reaction rate; however, precise control over the reaction is required to retain the fine features during the conversion^5^. In liquid phase conversion, the solvent should only dissolve the MAX salt but not dissolve the HPs. In addition, adequately high vapor pressure and partial wetting ability of the solvent are necessary to enable complete evaporation and diffusion inside the pores of the particles, respectively. Therefore, IPA is selected as an appropriate solvent^1^. Figure 4a shows the XRD results of anion IER versus reaction time from 5 min to 24 h. Small characteristic peaks of MAPbBr_3_ start to appear after 5 min of reaction and the peaks intensities gradually increase over reaction time as more PbCO_3_ are converted into MAPbBr_3_. However, the reaction is not complete even after 24 h, representing the slower rate as well as higher complexity of anion IER. To prove the formation of MAPbBr_3_, converted MAPbBr_3_ samples at different IER times were excited by a laser with 405 nm wavelength, and the emission of the particles was measured (Fig. 4b). Photoluminescence (PL) intensities of the converted MAPbBr_3_ samples increase with the rising of the anion IER time up to 30 min, resulting from the formation of more MAPbBr_3_ on the surface of the particles, then decrease by a further increase of IER time. The position and full width at half maximum (FWHM) of PL peaks are about 535 and 25 nm, respectively, which are consistent with optical properties of stoichiometric MAPbBr_3_ compound and indicate the full conversion of BaCO_3_ samples into MAPbBr_3_ nano/microstructures (Fig. S10). However, 15 nm redshift in PL spectra is observed over IER time. This might come from the fact that at a shorter IER time (≤ 30 min), the formed HP layer on the surface is very thin (in nanoscale range), and it progressively becomes thicker until it forms a bulk HP layer. SEM results depict the microstructures of the original BaCO_3_ and converted PbCO_3_ and MAPbBr_3_ samples (Fig. 4c). All morphologies resemble the original BaCO_3_ shape, offering effective conversion from metal carbonates into halide perovskite compound with preserved morphologies.

Figure 4d and S11 show EDX elemental mappings of converted MAPbBr_3_ samples with different morphologies at different anion IER times. Uniform conversion into MAPbBr_3_ composition is shown by the homogenous distribution of Pb and Br ions all over the surface of the samples. In addition, Ba ions (in the core) are also present due to the core–shell form of structures resulted in IER. The formation of core–shell structures results from the IER initiated at the surface of metal carbonate particles and from the fact that Pb^2+^ ions (and later CH_3_NH_3_^+^ and Br^-^ ions) diffuse into BaCO_3_ particles gradually over time. However, transformation inside BaCO_3_ particles is more challenging, and if the reaction time is too short, some BaCO_3_ particles may still exist in the core. The remaining BaCO_3_ is not problematic as BaCO_3_ is in the samples' inner parts (core). It can also be helpful to maintain the original morphologies by functioning as a scaffold and prohibiting the destruction of the 3D architectures^40^. In terms of applications, the existence of BaCO_3_ core is neutral as in most applications such as sensing properties of HPs, the surface properties of the samples determine the performance.

Interestingly, although the most obtained CaCO_3_ samples were in calcite form, they all could be converted effectively into MAPbBr_3_ nano/microstructures. Fig. S12 represents green light emissions under UV light irradiation of successful conversion of CaCO_3_ samples into MAPbBr_3_ synthesized with starch as bio-template at different temperatures and precursors concentrations. EDX elemental mappings of two converted MAPbBr3 samples with different morphologies reveal the uniform distribution of Pb^2+^ and Br- ions together with Ca^2+^ (from the core of the converted particles) while the original morphologies are well maintained (Fig. S13a). However, as the synthesized CaCO_3_ nano/microstructures have calcite type of atomic structure (hexagonal crystal system), the conversion into halide perovskite with the orthorhombic unit cell is more challenging, and the chance of structural distortion is higher compared with when the unit cell of metal carbonate is also orthorhombic (i.e., aragonite polymorph). Therefore, there are more possibilities to deviate from uniform conversion, and some other particles might start to form during the ion exchange reactions due to dissolution of metal carbonates and subsequent nucleation and growth of new particles on the surface (Fig. S13b). However, appropriate conversion reaction kinetics by using optimized concentrations of conversion solutions as well as reaction time and temperature can minimize the formation of new particles and assist the preservation of original morphologies.

The shape-preserving mechanism during ion exchange, complete chemical and structural transformation, rearrangement of the crystal unit cell, and minimum distortion of the crystal lattices are critical requirements for the successful conversion. In the first step, PbCO_3_ forms by replacing metal cations with Pb^2+^ ions within the carbonate material via cation IER. Higher thermodynamic stability of the resulting PbCO_3_, similar orthorhombic crystal structures of both MCO_3_ and PbCO_3_ as well as high concentration of Pb^2+^ and minimum dissolution- recrystallization of MCO_3_ guarantee the fast and complete conversion with minimized distortion of the unit cell. Secondly, the resulting PbCO_3_ architectures are converted directly into the HPs in a one-pot transformation via exposing the PbCO_3_ microstructures to a high concentration of dissolved CH_3_NH_3_X to push the reaction towards the consumption of the intermediate PbX_2_ phase and the formation of the HPs at room temperature^1,5,72^. This is a more challenging step due to the nature of anionic IER that anions are larger species with lower diffusivity. Fortunately, the carbonate anion can perform as a good leaving group for introducing the desired halide, and the intermediate PbX_2_ phase crystal unit cell resembles that of PbCO_3_ and many other carbonate compounds MCO_3_ (orthorhombic crystal structure). PbX_2_, PbCO_3_, and MCO_3_ possess the same metal ions crystal positions and relatively similar unit cell dimensions, thus facilitating the IER and maintaining the 3D morphology, fine features, and crystallinity of the preliminary structure. Moreover, the negative Gibbs free energy of formation of acquired HPs in comparison with the employed precursors and PbX_2_ spontaneously drives the transformation process^1,5,40,72^. Apart from the aforementioned parameters, other important control considerations during the entire IER procedure include pH, temperature, and the molar ratio of halides and organic cations^5,40,73–75^. The presented results confirm that many carbonate micro/nanostructures can be initially produced and subsequently converted into HP materials.

**Figure 5:**
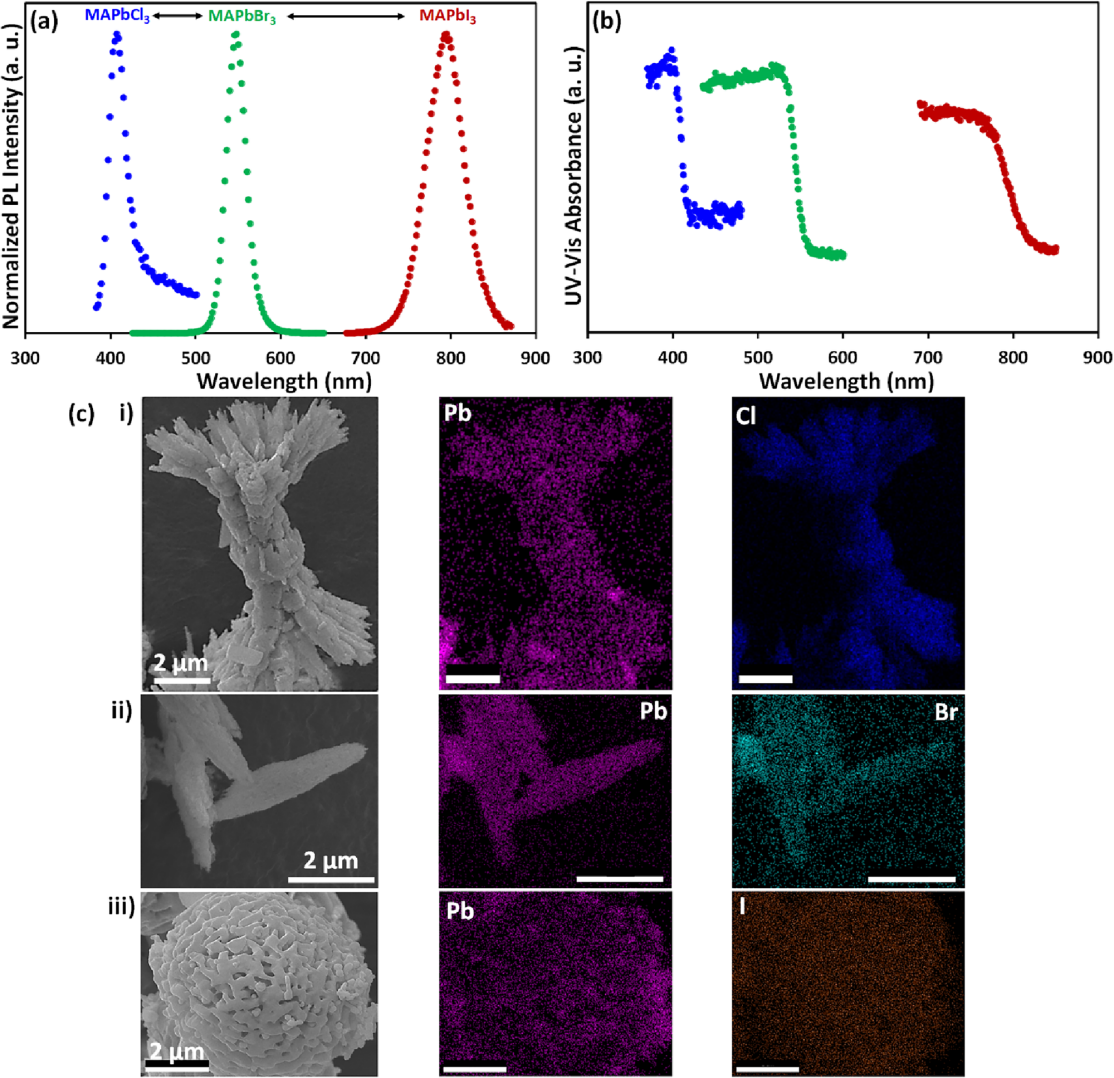
Conversion of nano/microstructures with different perovskite compositions. (**a**, **b**) PL and absorption spectra of MAPbCl_3_, MAPbBr_3_, and MAPbI_3_ samples obtained by IER of metal carbonate nano/microstructures. (**c**) EDX elemental mapping of converted (i) MAPbCl_3_, (ii) MAPbBr_3_, and (iii) MAPbI_3_ samples using M13 bacteriophage, BSA, and starch, respectively (scale bars = 2 µm).

To establish the versatility of our method, we converted metal carbonate samples into HPs with different halide anions to produce MAPbI_3_ and MAPbCl_3_ beside the MAPbBr_3_ compositions (Fig. 5 and S14). Results show that HPs with different halide groups and various emission colors can be formed by selecting the corresponding MAX precursor in IPA solution. The emission wavelength as well as FWHM of MAPbCl_3_, MAPbBr_3_, and MAPbI_3_ compositions are 406, 25, 546, 25, 792, 50 nm, respectively, consistent with previously reported characteristics. Consequently, the color of the light-emitting architectures, together with their band gaps, can be adjusted over the entire visible range while the original structure is well-preserved.

### Device fabrication

Despite the instability of HP compounds against environmental factors, i.e., oxygen, illumination, moisture, temperature, etc., it has been shown to be beneficial for sensing applications. In this regard, a number of gases, metal ions, pesticides, organic molecules as well as temperature sensors based on HP materials have already been manufactured^76^. Chen et al. developed a visible-light photoelectrochemical biosensor for the detection of dopamine based on TiO_2_/CsPbBr_1.5_I_1.5_/Nafion electrode from 0.1 to 250 μM^77,78^. Maity et al. ^79^ has recently innovated a paper-based color change visual sensor made up of CH_3_NH_3_PbI_3_ as the active material to detect ammonia gas. The mechanism involved is the color change of black CH_3_NH_3_PbI_3_ film on the paper to yellow color due to the decomposition of CH_3_NH_3_PbI_3_ to PbI_2_ when exposed to meager amount of NH_3_ gas (≅ 10 ppm) with a fast reaction time of approximately 10 s. The same group has later manufactured an electronic paper-based solid-state NH_3_ gas sensor with sub ppm sensitivity better than 1 ppm, using the same principles as the previous visual color change gas sensor^80^.

**Figure 6:**
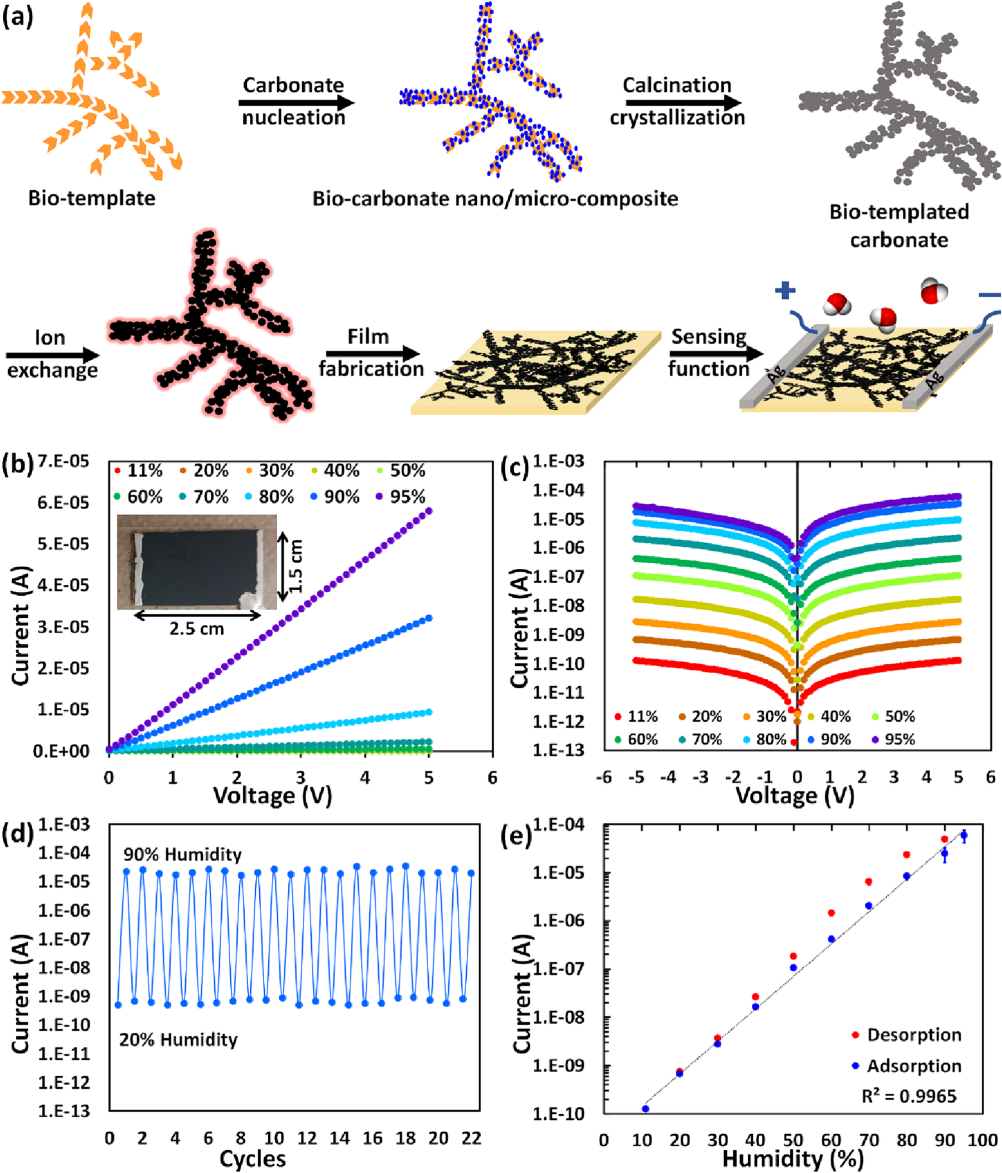
Fabrication of thin film humidity sensors using bio-templated MAPbI_3_. (**a**) Schematic illustration displaying the construction steps of a disposable paper-based sensor from the 3D shaped MAPbI_3_ nano/microstructures. (**b**) I–V characteristics of the fabricated paper based CH_3_NH_3_PbI_3_ device as a function of humidity in linear scale. The inset shows the top view of fabricated paper-based device with actual dimensions. (**c**) Logarithmic representation of the I–V data over different humidity conditions. (**d**) Multiple cycles of sensing ability of the fabricated device for humidity range between 20 and 90%. (**e**) Device current as a function of humidity levels and reversible adsorption–desorption behavior, showing high linearity with R^2^ = 0.9965.

We also believe our approach helps expand the use of HP 3D assemblies not only for the HPs conventional applications but also for new usages such as biological and environmental applications. As proof of concept, we fabricated a disposable thin film device by simply casting HP nano/micro-structures on paper substrates to demonstrate the beneficial and practical capabilities of the as-prepared HPs 3D nano/micro-composites. Our approach for constructing the paper-based humidity sensor is outlined in Fig. 6a. To prepare the humidity sensing device, we applied the assembled 3D nano/microstructures with CH_3_NH_3_PbI_3_ composition on a paper substrate by simple drop casting method from the IPA solution at the last conversion step, followed by rinsing with IPA to wash it out. The fast IPA evaporation helps the deposited film quickly dry, and the porosity of the paper substrate assists appropriate attachment of CH_3_NH_3_PbI_3_ nano/microstructures. Finally, electrodes are created by applying silver paste on both ends of the device by hand.

As depicted in Fig. 6b,c, the conductivity of the CH_3_NH_3_PbI_3_ paper sensor continuously increases as a function of humidity rise in the wide range of 11–95%, which is reasonably comparable with other HP based humidity sensors (Table S1). The considerable increase of more than 5 orders of magnitude of the current when the humidity increases from 11 to 95% implies promising moisture adsorption capability as well as strong moisture-induced effect on the charge transport characteristics of the sensor. The stability and reproducibility of the device are shown in Fig. 6d by cyclic measurement of the film current upon exposure to humidity conditions between 20 and 90%. Figure 6e shows the variation of current values on a logarithmic scale at a potential bias of 5 V with a linear performance (coefficient of determination R^2^ = 0.996) throughout the studied range, offering a viable humidity-sensing range. In addition, the current values are measured during the desorption process, which demonstrates the formation of a small hysteresis between adsorption and desorption steps. This is due to the binding of adsorbed water molecules with the HP particles as well as pores and capillaries, which require a longer time for dehydration processes to take place. The excellent long-term stability of the sensor is determined by relatively steady current values over two months at 35% ambient humidity (Fig. S15), indicative of a daily life sensing application.

According to previously reported papers, different hypotheses are proposed to explain how water adsorption causes conductivity enhancement of HPs. For instance, it has been suggested that water molecules on the surface act as electron acceptors and improve the HPs conductivity or introduce new states in the HPs band gap by triggering lattice distortion that boosts the conductivity^81,82^. However, most recently, Ilin and coauthors^83^ explained that both ionic and electronic modes of conductivity are concurrently possible at room temperature. At low humidity conditions (water vapor pressure below 10 mbar), electronic conductivity is dominant by passivating dangling bonds on the surface of HPs. At higher humidity, more than one monolayer of water molecules may be adsorbed in the pores, helping protons mobility, resulting in domination of ionic conductivity^83–88^.

There is strong evidence that sensing and catalytic performances of several types of materials can be strongly dependent on particles morphology. Desirable morphologies to provide high sensitivity are required to have high purity, good crystallinity, and preferably porous microstructure, which are advantageous for electron transfer. Besides, nano/microfeatures of the desired shapes have significant influence on the surface area, active sites, and charge carrier kinetics of materials, which can efficiently impact on sensing performance. A large specific surface area is often essential to provide more active sites, absorb more analytes, and permit the electrons to transit inside the pore cavities, hereafter accelerating the sensing activities. For example, the nanoscale morphologies of the CH_3_NH_3_PbI_3_ perovskite films can strongly affect the device sensitivity to O_2_ gas^89^. Recent report showed that the dimensionality of CsPbBr_3_ perovskite films have also large impact on sensing ability to common explosive vapors as external stimuli. Response of the films made of nanocrystals was larger and faster than their 2D and 3D counterparts because of less dense film, easier pathways for the vapor to percolate into the film, much larger surface area, and increased exciton binding energy^90^. Humidity is a physiochemical phenomenon and the capability of an active material to sense the presence of moisture mostly depends on the reactivity of its surface, i.e., the accessibility of adsorption sites and the interaction strength between the adsorbate and the water molecules. The reactivity of the surface relies on the surface chemistry (cation/anion distribution) and morphology. Different morphologies like rods, rod-flowers, flakes, and flake-flowers were revealed to alter the electrical, optical, and sensing properties of nanostructured SnSe samples^91^. The rod morphology had the best humidity sensing performance with high sensitivity, good repeatability, low hysteresis in the absorption–desorption process, and good reproducibility. Hexagonal morphology of CoFe_2_O_4_ nanoparticles demonstrated higher sensitivity and faster response/recovery time for humidity sensing in comparison with spherical, and cubic nanoparticles^92^. Likewise, the performance and response time of our humidity sensor is improved in the order of branched-porous > elongated rod > cubic morphologies, conceivably due to their active surface area, accumulation ability for humidity, and better charge transfer rates (Fig. S16a). However, all morphologies eventually reach the same sensitivity range. Furthermore, the stability of the built humidity sensor strongly depends on the morphology of the particles used to create the perovskite film. Figure S16b demonstrates that the film composed of more hierarchical porous nano/microstructures exhibits the greatest stability over a period of two months, while the film formed from large cubic particles (i.e., no template used for the synthesis) shows the least stability, with significant performance fluctuations after about two weeks.

Our homemade device dimensions (1.5 × 2.5 cm) are much bigger than similar ones reported previously^83,87^, demonstrating the straightforward scalability of our approach (Fig. 6b). To show the versatility of our device, we deposit CH_3_NH_3_PbBr_3_ HPs on paper, and the I–V curve is presented in Fig. S17. Furthermore, we subjected our paper-based sensor to ammonia gas to authenticate its power for biological usages. It shows conductivity increases more than 2 orders of magnitude upon exposure to ammonia gas on par with previously reported sensors (Fig. S18)^93–95^.

Overall, this prototype design already proves its potential for real-life optoelectronic applications. Our device performance may be further improved by providing better attachment between the HP particles themselves together with the electrodes. Intriguingly, our paper-based nano/microstructured gas sensor can be introduced to the next-generation smart biosensors with several advantages: rapid and selective detection, high sensitivity and instant response to biological molecules, large linear range of detection, simple construction, low cost, simple operation, disposability, to name a few. Also, complex 3D assembled morphologies provide high surface area, maximizing detection and improving charge transport for higher sensitivity and performance. Last but not least, most conventional sensors require operating at high temperatures, having low selectivity, slow response/recovery rate, and poor detection limit^96,97^, but our device can work at room temperature with no need for an external power supply.

## Conclusion

In summary, we combine versatile bio-template and biomineralization techniques with morphology controlled IER approach to assemble naturally inspired complicated HP 3D geometries with nano/micrometer accuracy. Structural and elemental analyses confirm the chemical transformations with conservation of the nano/microscopic morphologies and nanocomposites layout. We showed the reaction time is critical on conversion and structural evolutions during both cation and anion IER steps, which has not been investigated before for HPs. We find that the nanocomposite layout also supports the formation and shape preservation of intermediate phases (e.g., PbCO_3_ and PbX_2_) and final HP products. Despite the control over the 3D structures, we also tuned the chemical composition of the naturally inspired HPs and constructed a paper-based thin film prototype device for environmental applications such as humidity and gas sensing. Therefore, this approach bestows biologically inspired materials with desirable combinations of characteristics and eventually enables applications ranging from potent catalysts and sensors to high-performance optoelectrical devices.

## Materials and methods

### Precursors

All precursors were directly used as received without further modifications or purifications. BaCl_2_ (99%, ACP Chemicals), CaCl_2_ (99%, ACP Chemicals), Na_2_CO_3_ (99%, ACP Chemicals), Pb(NO_3_)_2_ (99%, Acros Organics), HI (48%, ACP Chemicals), HBr (48%, Sigma-Aldrich), HCl (36–38%, Sigma-Aldrich), Methylamine solution (CH_3_NH_2_, 40 wt.% in water, Sigma-Aldrich), Distilled water, isopropanol (IPA, 99%, Bio-Basic), BSA (98%, BioShop), starch (98%, Sigma-Aldrich).

### Synthesis of CaCO_3_

To prepare for the CaCO_3_ synthesis using spontaneous precipitation method, aqueous solution of CaCl_2_·2H_2_O (20, 50 and 125 mM), Na_2_CO_3_ (20, 50, and 125 mM), soluble starch (0.25 wt%), and dispersed curli fibers (expressed from Escherichia coli bacteria and isolated from previously reported method^54,63^) suspension were first prepared as stock solution (0.25 wt%). Next, 40 ml of both CaCl_2_·2H_2_O and Na_2_CO_3_ solutions with the same concentration were taken from the respective stock solution and injected into two separate beakers. The same amount (40 ml) of bio-template solution was then injected to the beaker containing CaCl_2_·2H_2_O and kept under moderate stirring for 10 min. Hot plate and ice bath were used for carrying out synthesis under different temperatures (0, 25, 50, and 75 °C). To initiate the precipitation, Na_2_CO_3_ solution was quickly injected to the solution mixture while being stirred vigorously for another 1 min. Quickly after, the product solution was transferred into a conical tube to perform repetitive washing and centrifugation steps for removing soluble by products from the solution and collecting the CaCO_3_ particles formed. The white precipitates of CaCO_3_ were then dried in air and followed by calcination step in furnace at 450 °C for 30 min to fully crystalize the samples and remove the bio-templates.

### Synthesis of BaCO_3_

Despite some small alterations, the same procedure was employed for the fabrication of BaCO_3_ nanostructures under different temperatures (0, 25, 50, 75, and 100 °C) and by using soluble starch (0.25 wt%), M13 bacteriophage (1.66 × 1013 phages/ml, obtained from previously reported method^52,64^), and bovine serum albumin (BSA, 0.25 wt%) as bio-templates. For BaCO_3_ synthesis using M13 bacteriophage, the concentrations of both reactant solutions (CaCl_2_·2H_2_O and Na_2_CO_3_) were diluted to 2 mM, and 500 μl of M13 bacteriophage solution was used along with 200 ml of each reactant.

### Conversion of MCO_3_ to CH_3_NH_3_PbX_3_

Before converting CaCO_3_ and BaCO_3_ into the corresponding halide perovskites, they must be first converted into PbCO_3_ through cationic exchange. To do so, Pb(NO_3_)_2_ was first dissolved in water to form a Pb(NO_3_)_2_ solution with a concentration of 0.9M. The Pb(NO_3_)_2_ solution was then added to the MCO_3_ samples and incubated from 1 to 10 min at room temperature before washing the PbCO_3_ sample produced with water and acetone. Appropriate amount of CH_3_NH_3_X (500 mg) was dissolved in isopropanol (IPA) to obtain a CH_3_NH_3_X/IPA mixture with a concentration of 0.13 M. 50 ml of this mixture was then applied onto the converted PbCO_3_ from the previous step to produce final CH_3_NH_3_PbX_3_ by performing anion exchange reaction in liquid phase at different reaction times (up to 24 h).

### Characterization

X-ray diffraction (XRD) analysis was performed with a Bruker D8 Discovery X-ray Diffractometer (VANTEC Detector, Cu-Ka (alpha)). Scanning electron microscopy (SEM) imaging was carried out using a FEI Quanta 450 ESEM at 10 kV. SEM X-ray spectrometer (EDS) was used for the determination of elemental chemical composition. UV–Vis absorption and photoluminescence (PL) spectra were recorded with Thermo Scientific Evolution 300 UV–Vis and Horiba Flouromax-4 spectrophotometers, respectively. The I–V measurements we conducted using an Agilent B1500A Semiconductor Device Analyzer in a humidity-controlled chamber at ambient temperature (Fig. S19).

### Supplementary Information

Supplementary Information.

## Data Availability

Research data is shared if requested to the corresponding author.
