# Supplementary material for: Mimicking nature to develop halide perovskite semiconductors from proteins and metal carbonates
Source: Sci Rep. 2024 Jul 4;14:15357. doi: 10.1038/s41598-024-66116-8 (PMC11224268; doi:10.1038/s41598-024-66116-8)
Supplement: Supplementary file 1 — Supplementary Information. [file 41598_2024_66116_MOESM1_ESM.docx]

**Mimicking Nature to Develop Halide Perovskite Semiconductors from Proteins and Metal Carbonates**

Masoud Aminzare,^a^ Yangshixing Li,^a^ Sara Mahshid ^b^ and Noémie-Manuelle Dorval Courchesne ^*a^

^a^ Department of Chemical Engineering, McGill University, 3610 University Street, Wong Building, Room 4180, Montreal, QC, H3A 0C5, Canada.

^b^ Department of Bioengineering, McGill University, 817 Sherbrooke Street West, Macdonald Engineering Building, Room 355, Montreal, QC, H3A 0C3, Canada.

*Correspondence to: noemie.dorvalcourchesne@mcgill.ca


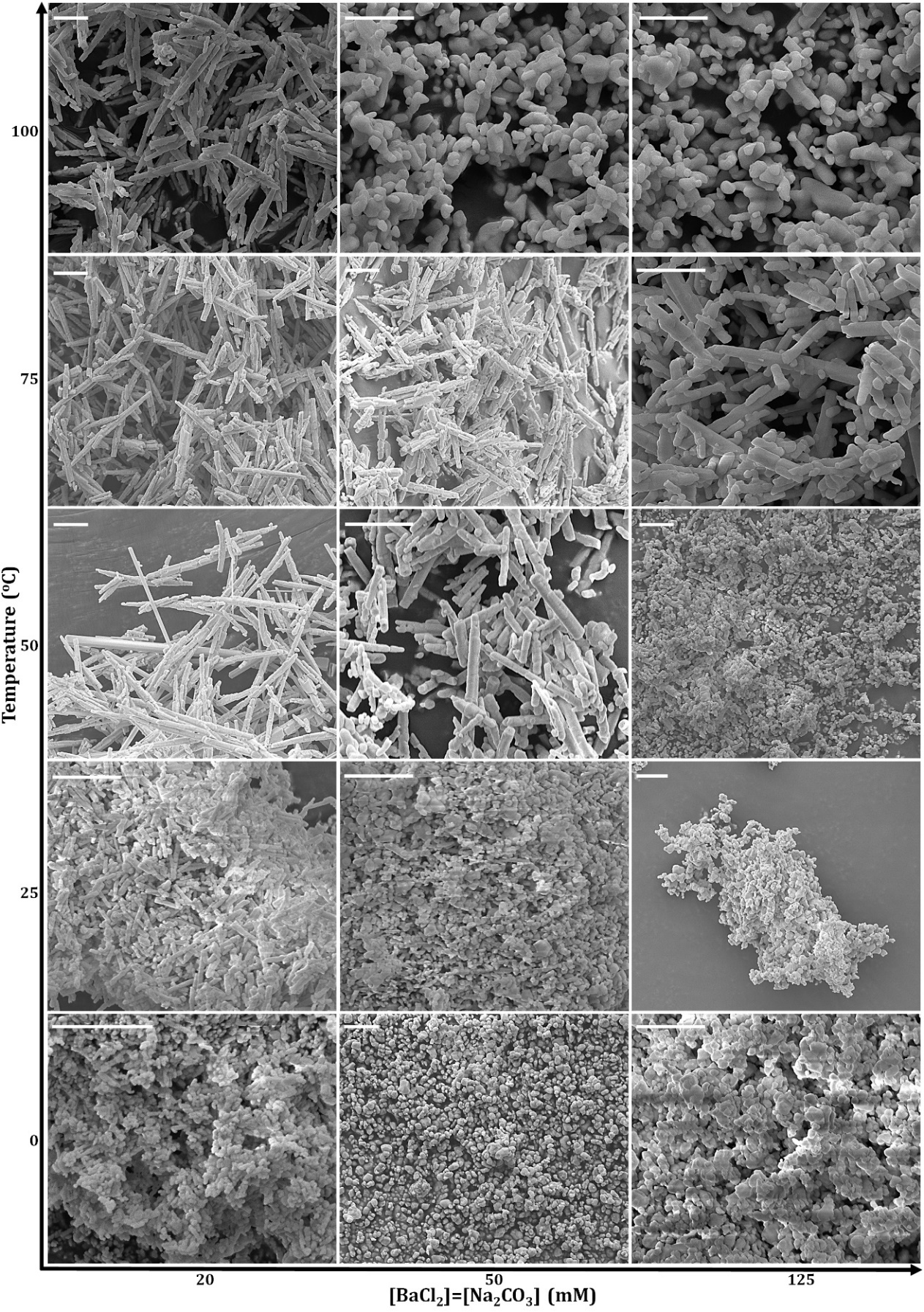


**Figure S1.** Effect of solution temperature and precursors concentration on the morphology of BaCO_3_ nano/microstructures synthesized with no bio-template (scale bars: 3 µm).


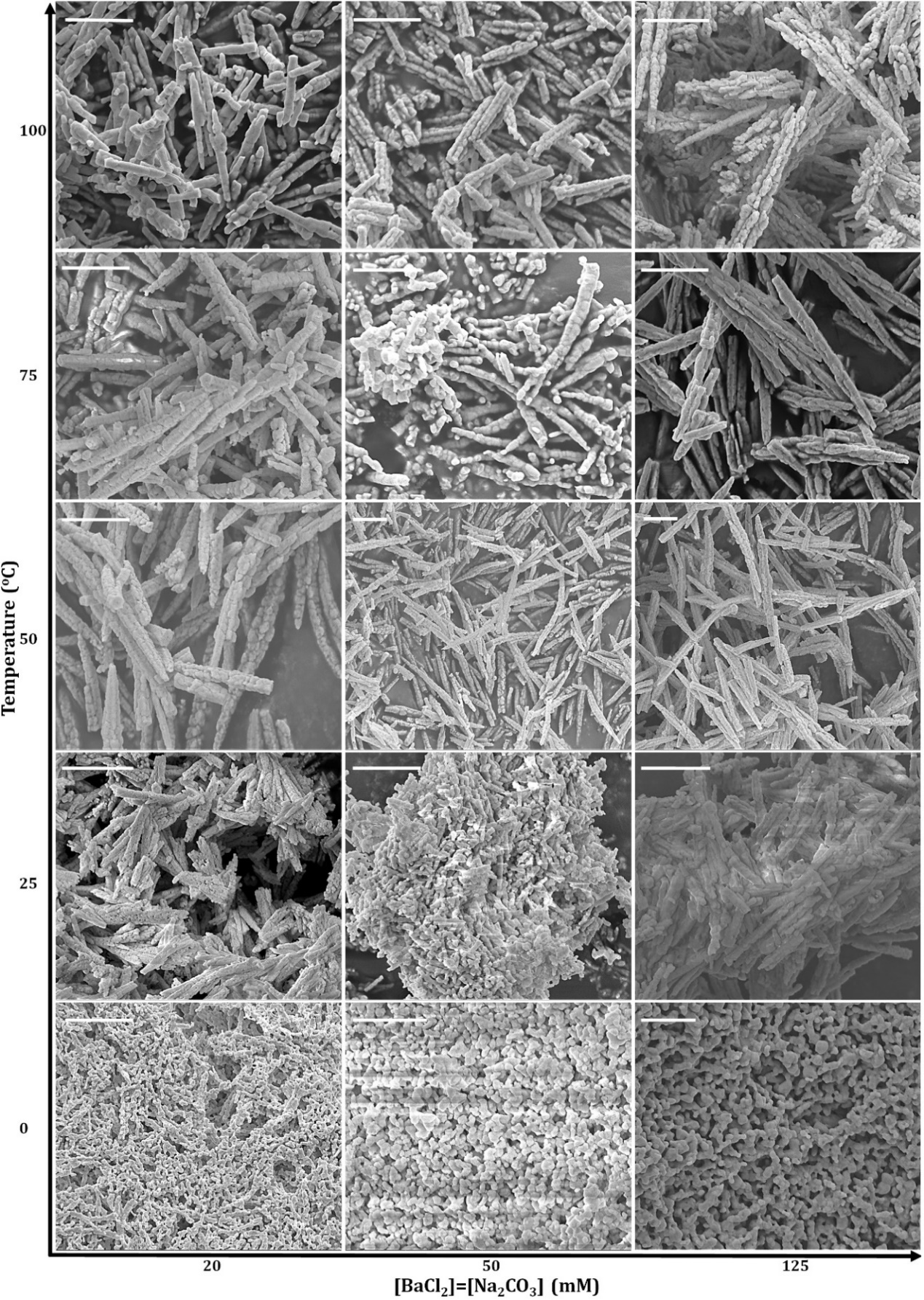


**Figure S2.** Effect of solution temperature and precursors concentration on the morphology of BaCO_3_ nano/microstructures synthesized with starch as bio-template (scale bars: 3 µm).


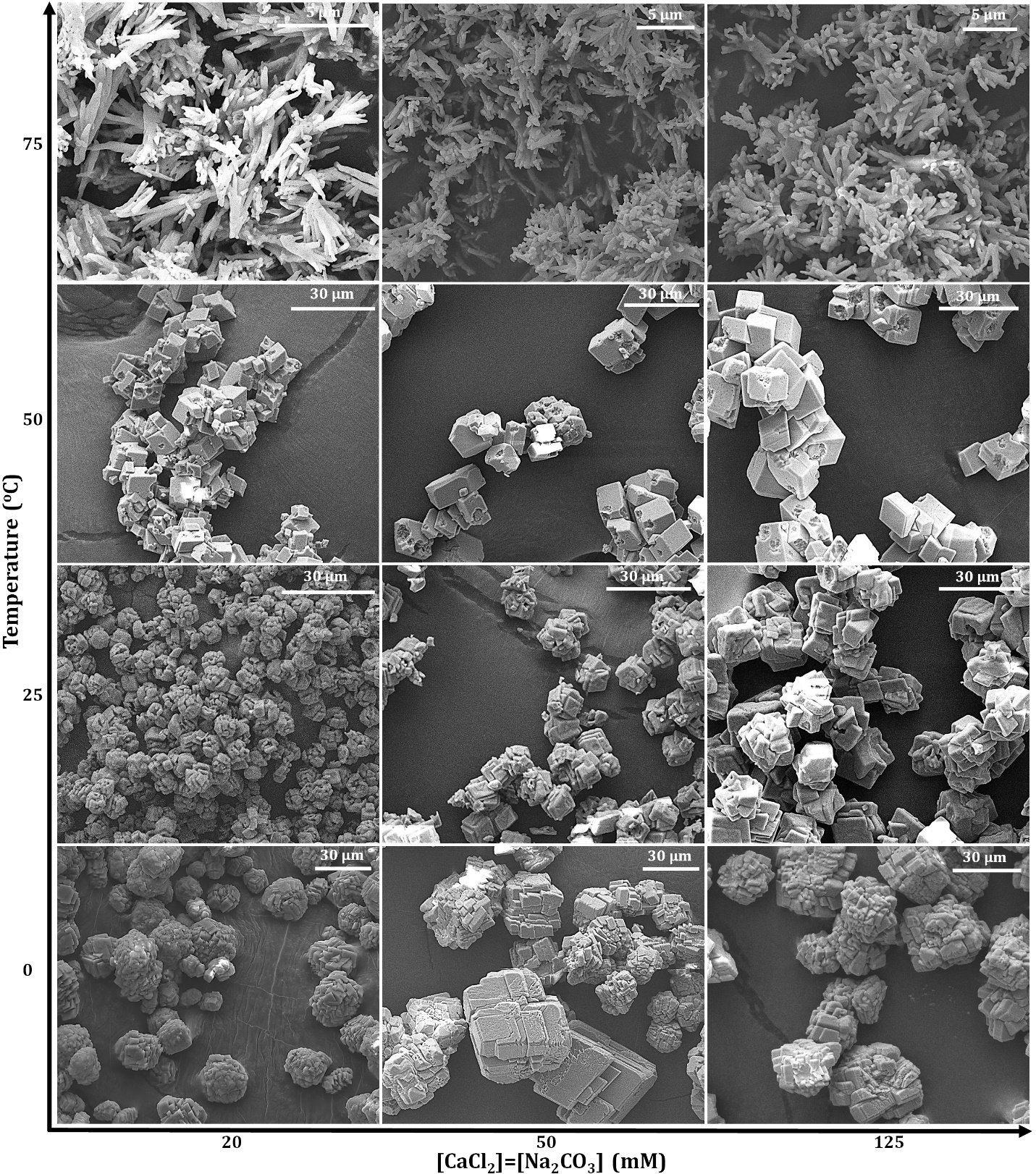


**Figure S3.** Effect of solution temperature and precursors concentration on the morphology of CaCO_3_ nano/microstructures synthesized with no bio-template.


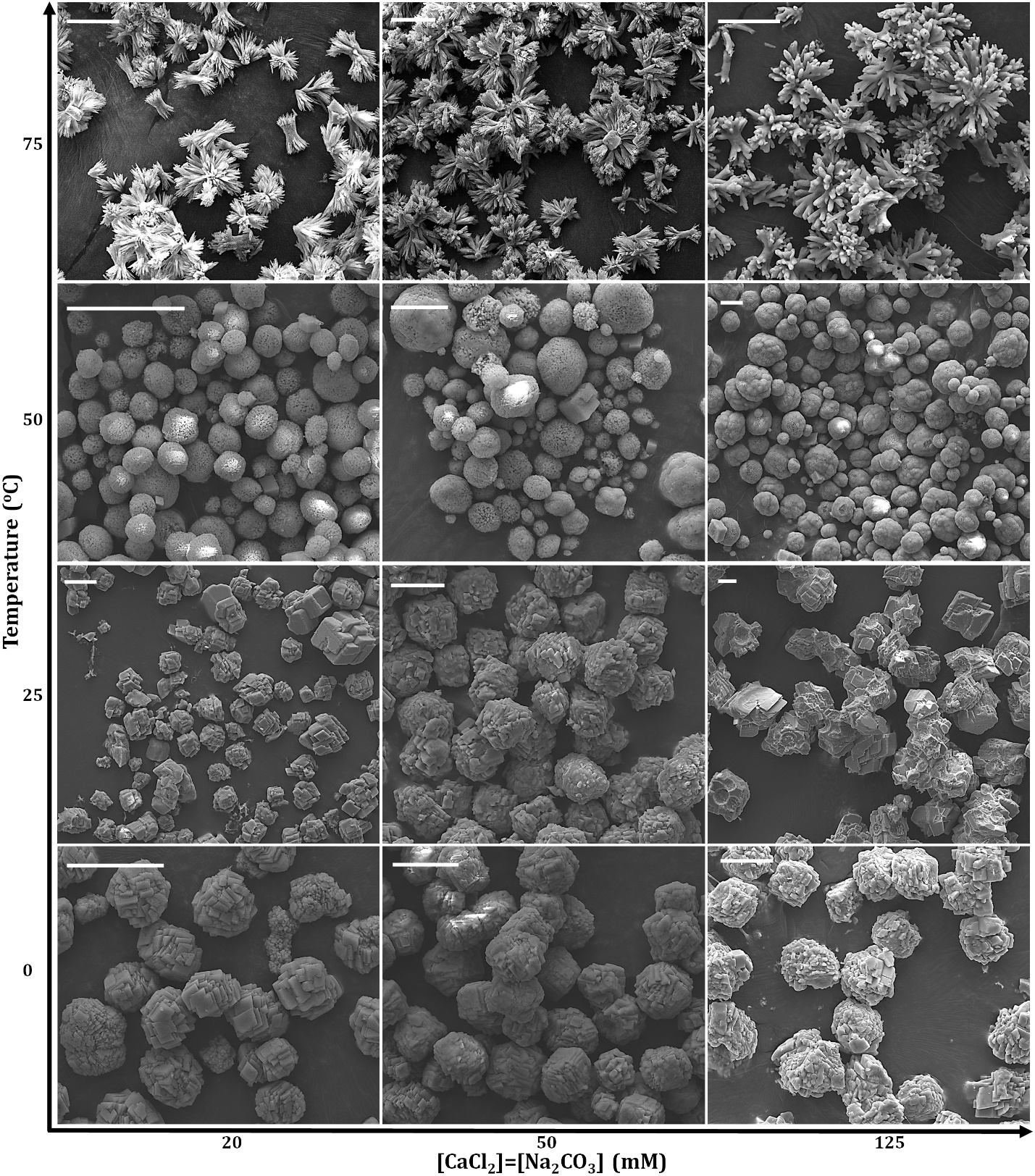


**Figure S4.** Effect of solution temperature and precursors concentration on the morphology of CaCO_3_ nano/microstructures synthesized with starch as bio-template (scale bars: 10 µm).


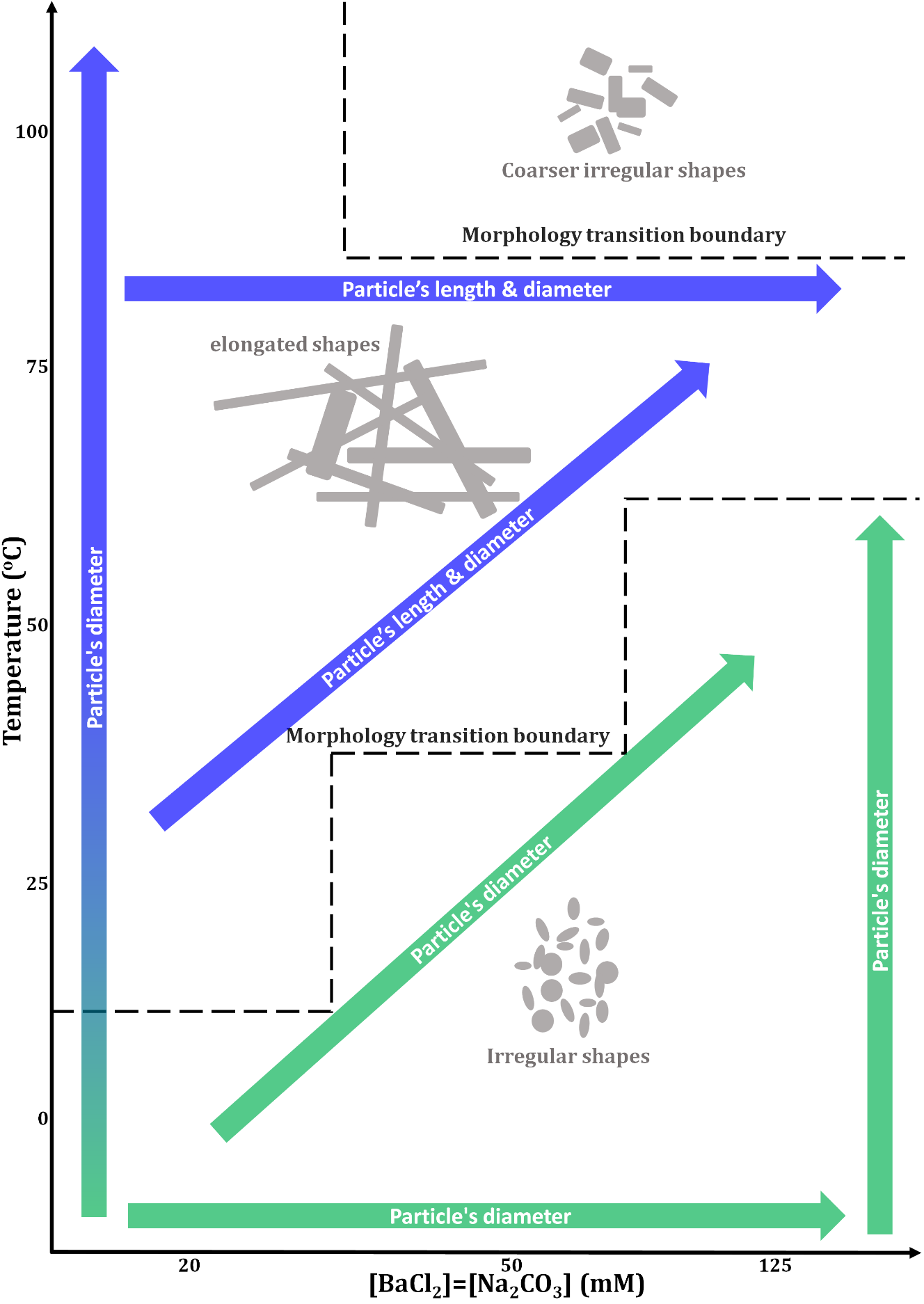


**Figure S5.** Schematic illustration of the effect of solution temperature and precursors concentration on the trend of morphology changes of BaCO_3_ nano/microstructures synthesized with no bio-template.


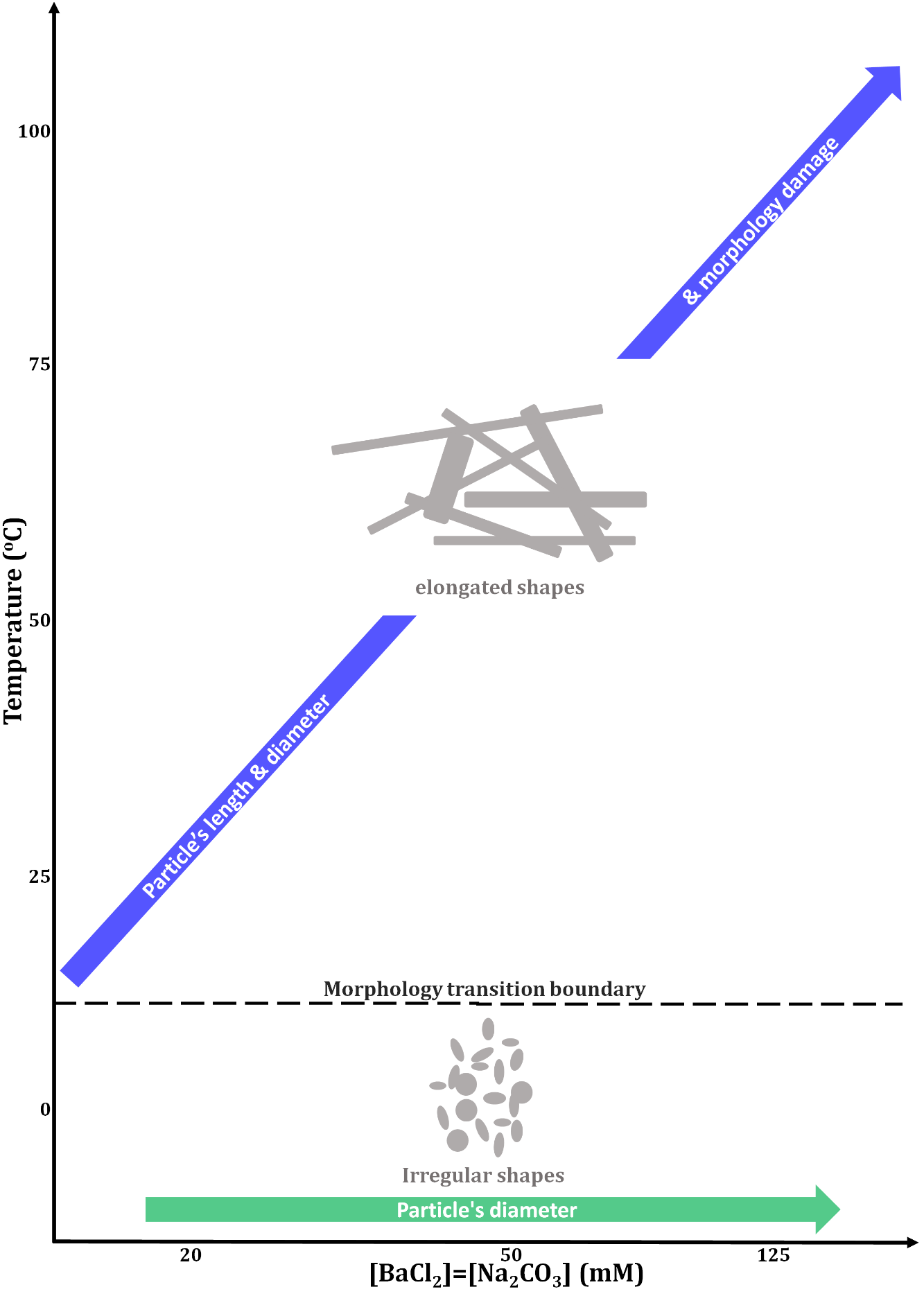


**Figure S6.** Schematic illustration of the effect of solution temperature and precursors concentration on the trend of morphology changes of BaCO_3_ nano/microstructures synthesized with starch bio-template.


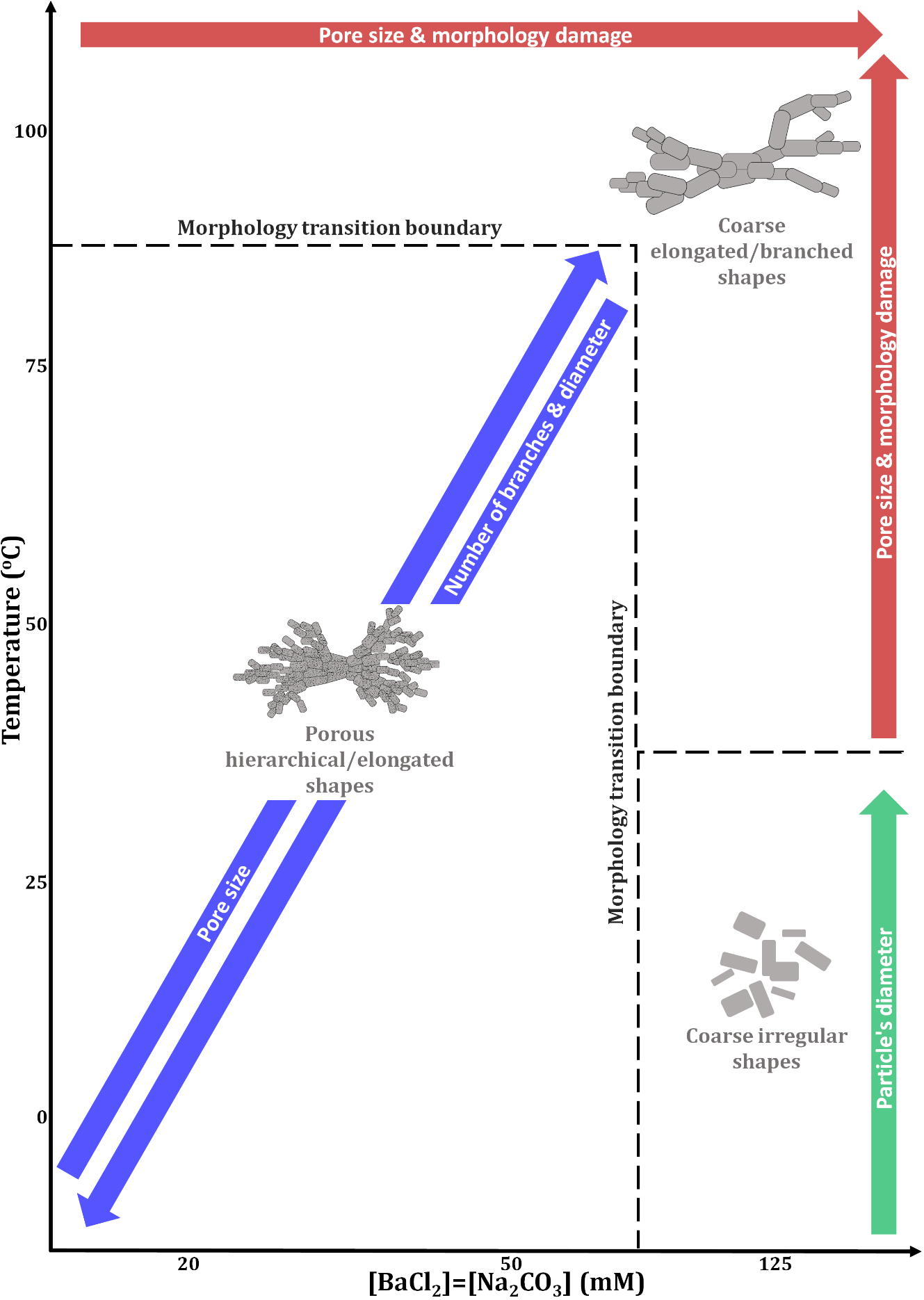


**Figure S7.** Schematic illustration of the effect of solution temperature and precursors concentration on the trend of morphology changes of BaCO_3_ nano/microstructures synthesized with BSA.


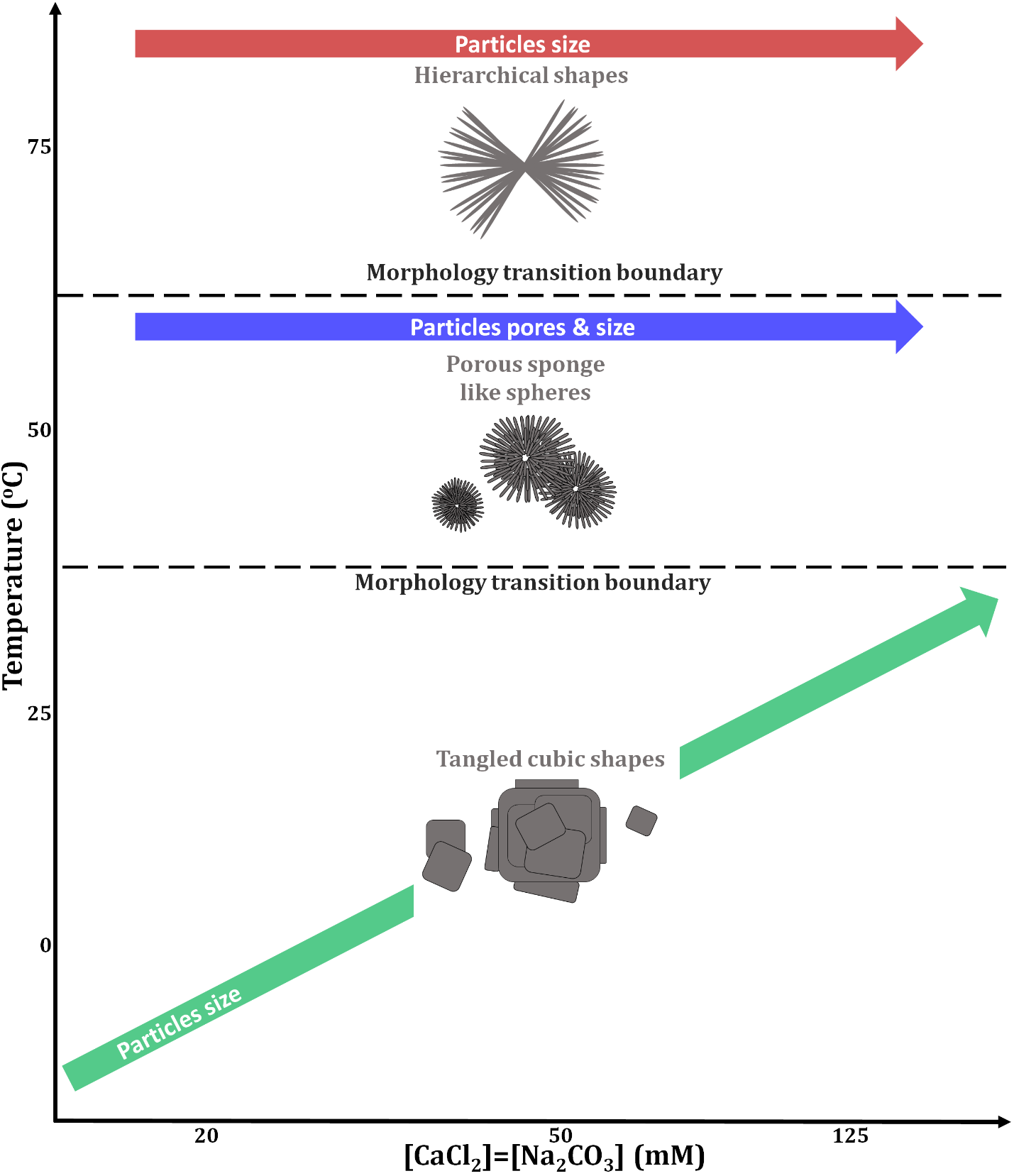


**Figure S8.** Schematic illustration of the effect of solution temperature and precursors concentration on the trend of morphology changes of CaCO_3_ nano/microstructures synthesized with starch bio-template.


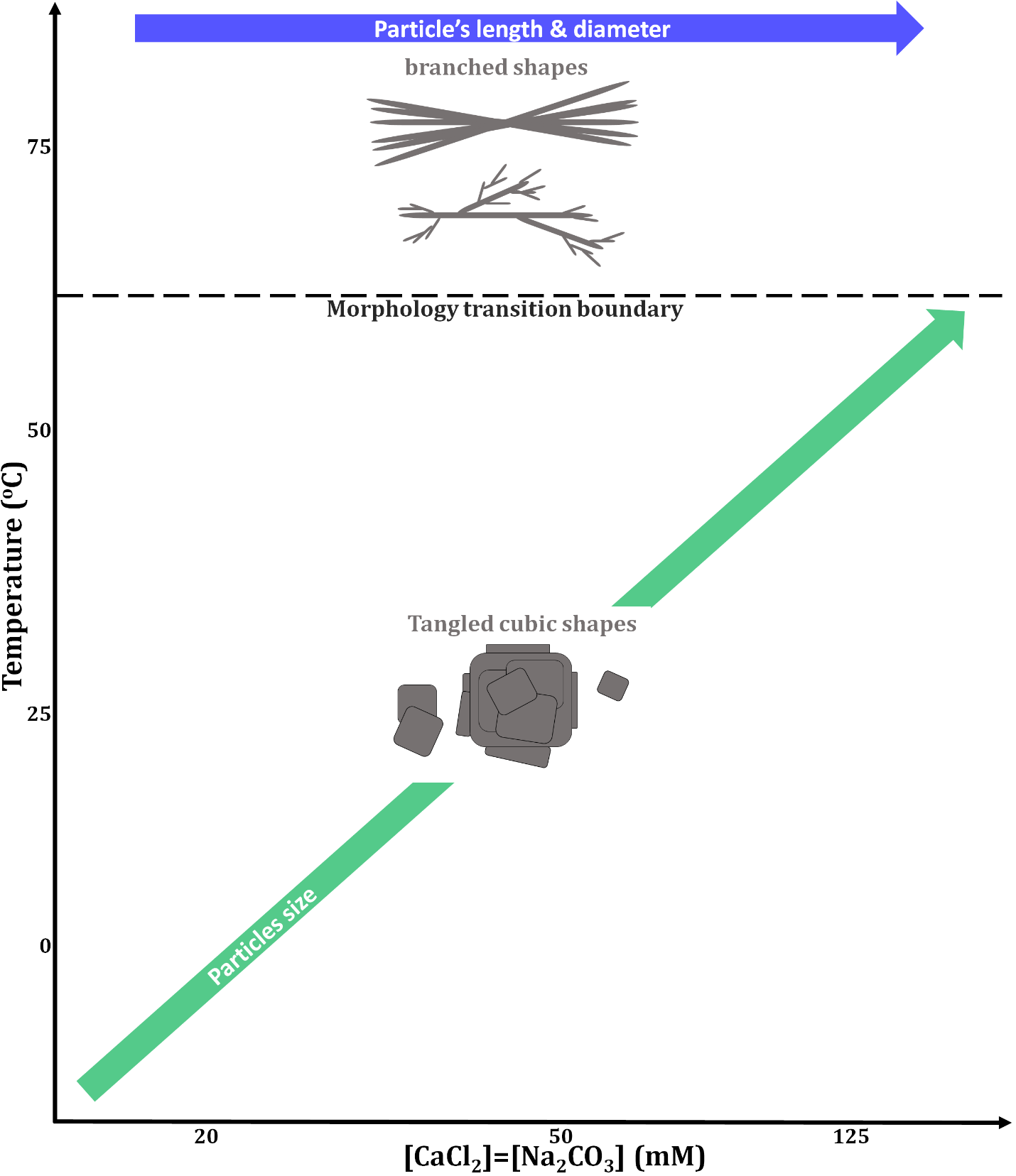


**Figure S9.** Schematic illustration of the effect of solution temperature and precursors concentration on the trend of morphology changes of BaCO_3_ nano/microstructures synthesized with no bio-template.


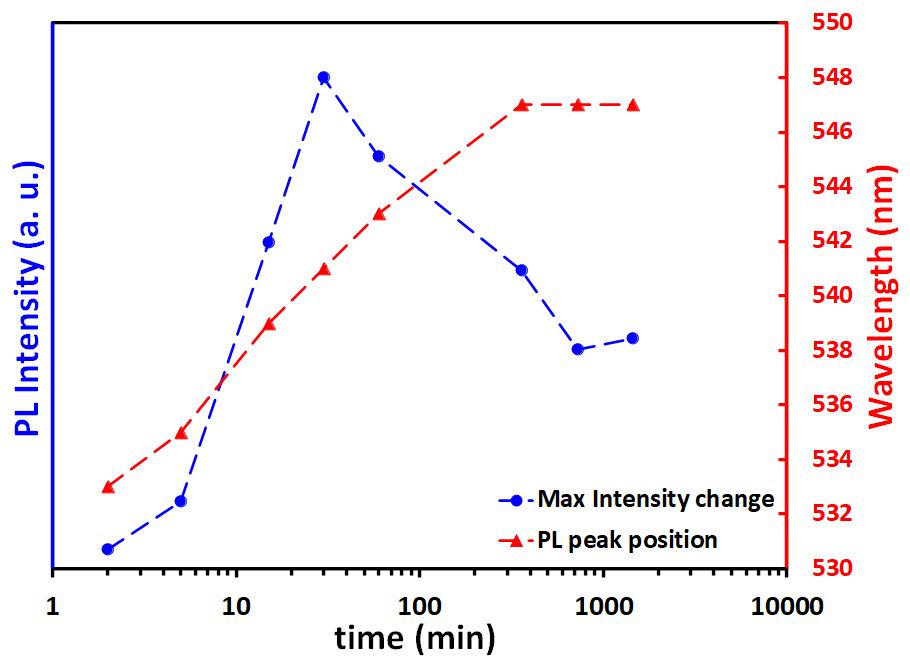


**Figure S10.** Max PL intensity and position of converted MAPbBr_3_ nano/microstructures as a function of IER time.


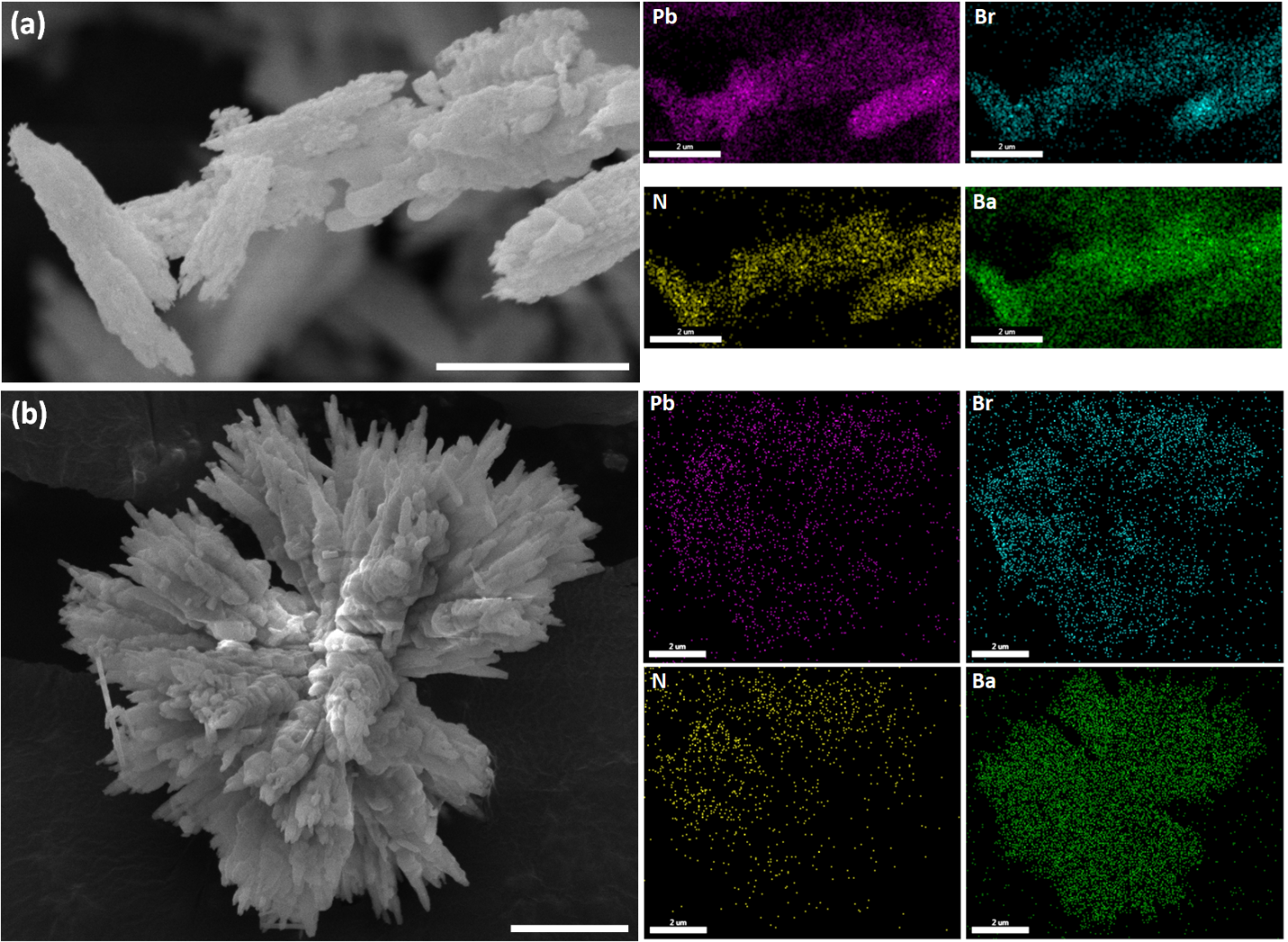


**Figure S11.** EDX elemental mapping of different converted MAPbBr_3_ nano/microstructures after (a) 60 (using BSA bio-template), (b) 15 minutes (using M13 bacteriophage bio-template) conversion reaction time (scale bars = 2 µm).


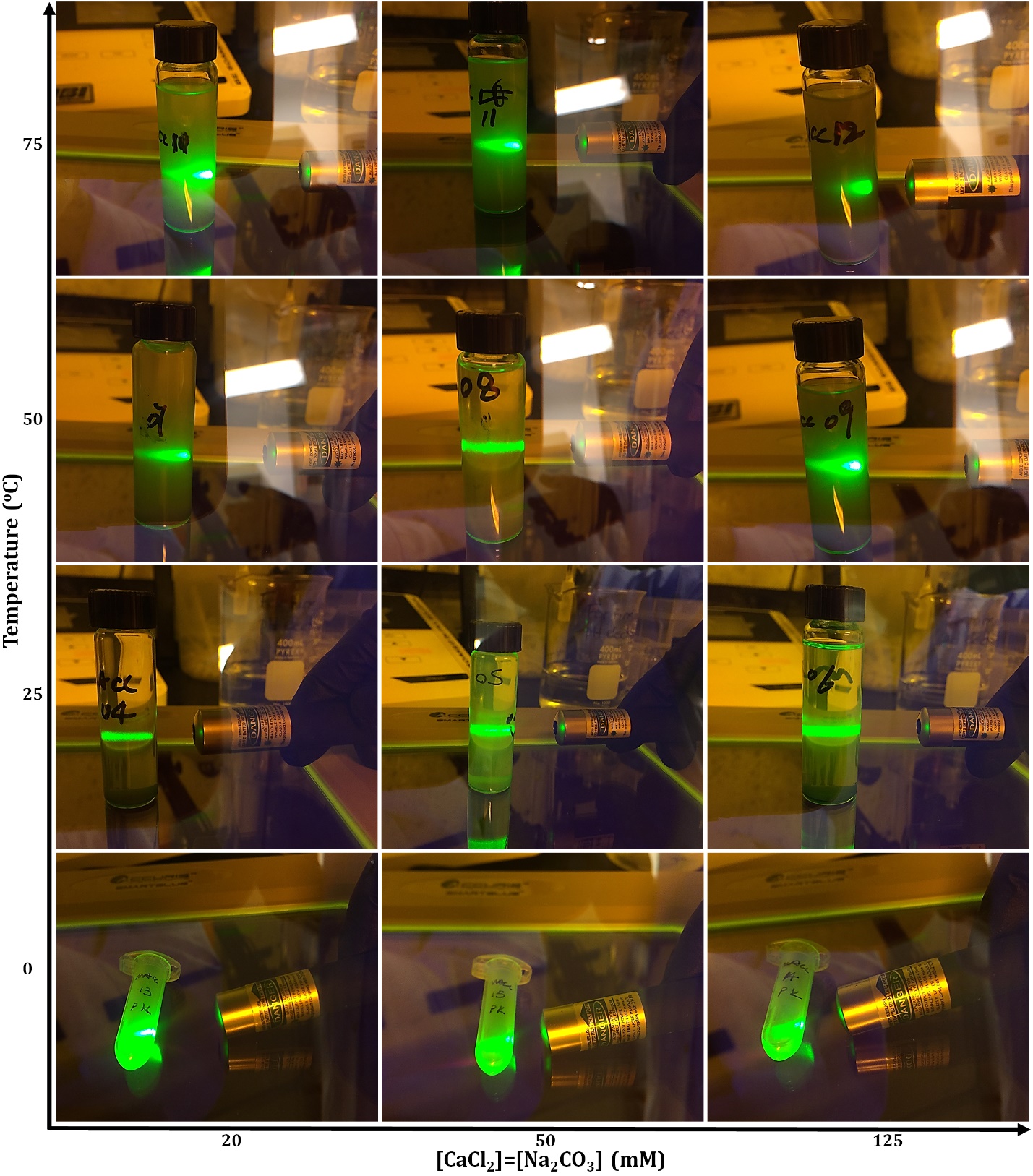


**Figure S12.** Green light emission of converted MAPbBr_3_ nano/microstructures under UV light irradiation synthesized with starch as bio-template at different solution temperatures and precursors concentrations.


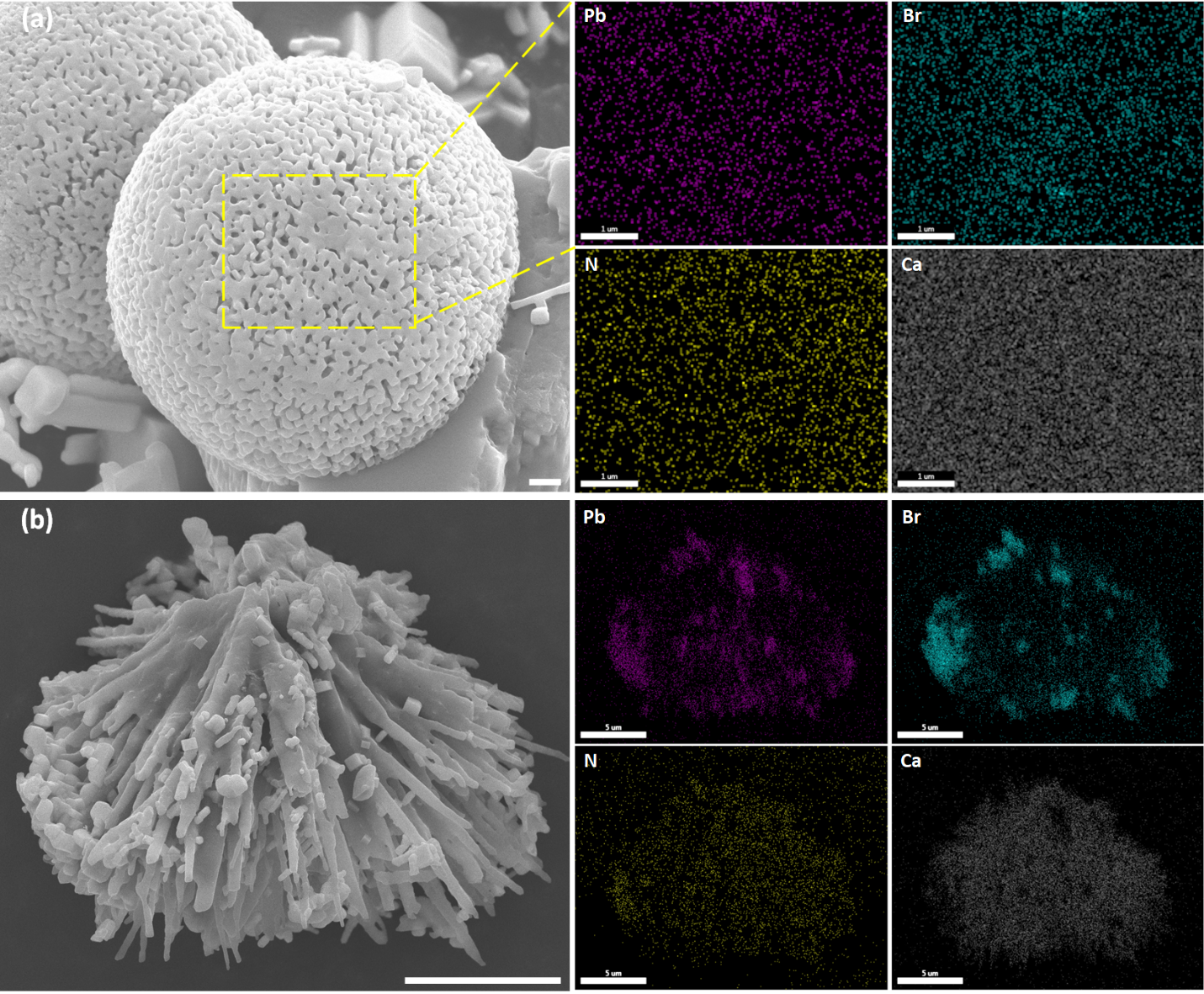


**Figure S13.** EDX elemental mapping of converted MAPbBr_3_ nano/microstructures from CaCO_3_ samples with different morphologies using starch as bio-template.


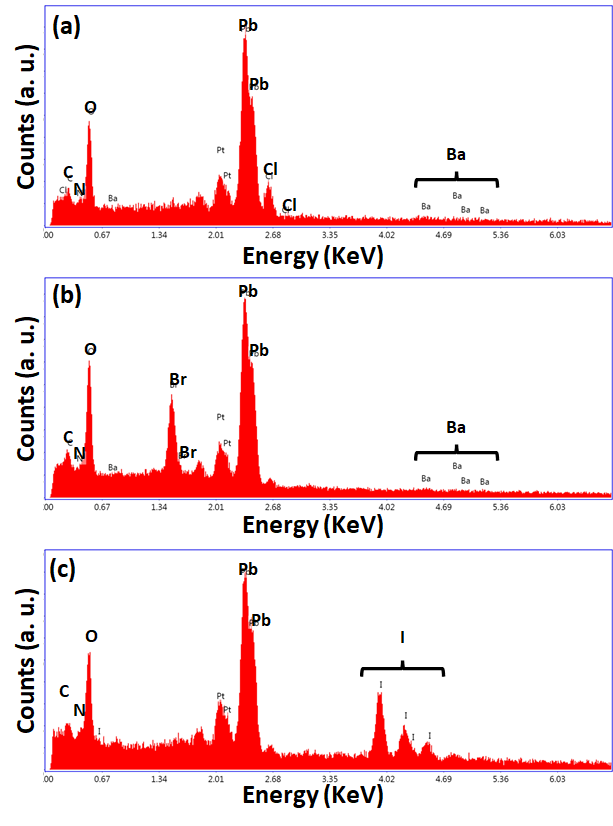


**Figure S14.** EDX elemental analysis of converted (a) MAPbCl_3_, (b) MAPbBr_3_, and (c) MAPbI_3_ samples using M13 bacteriophage, BSA, and starch, respectively.


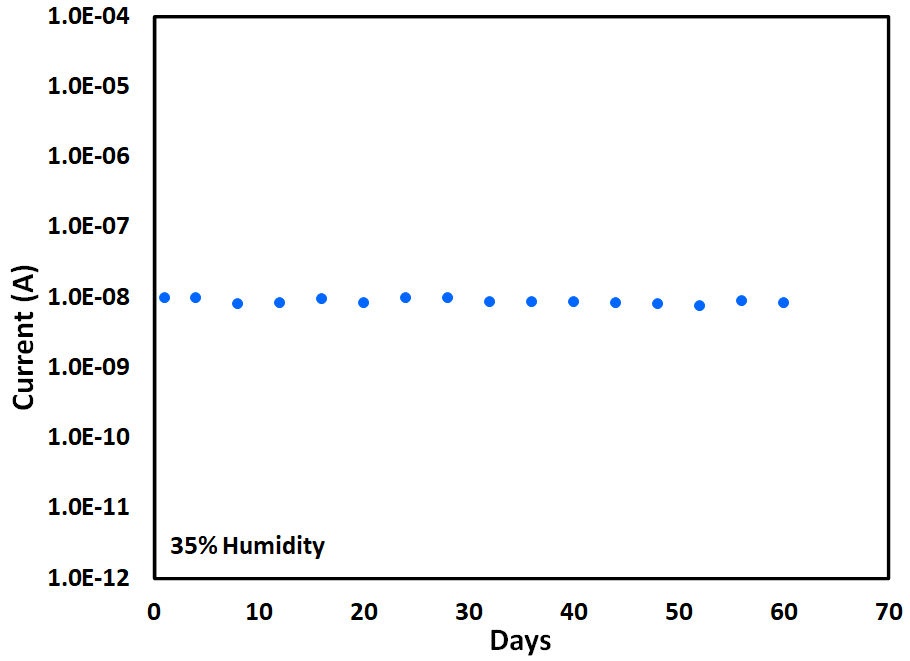


**Figure S15.** The consistency of sensor response at 35% humidity level as a function time in 2 months.


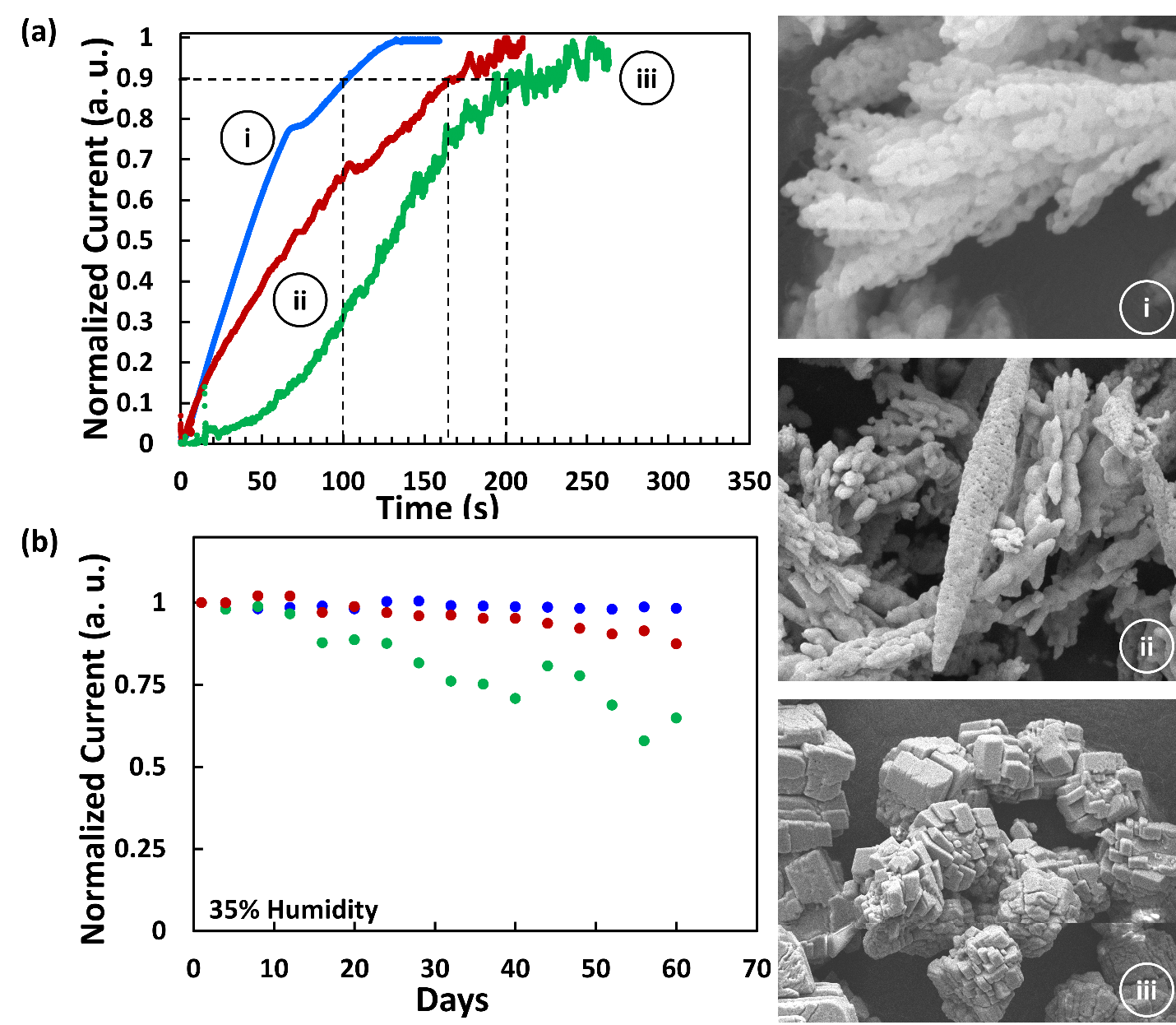


**Figure S16.** Performance, (a) response time and (b) stability, of humidity sensors made in this project based on three different morphologies. The longer response time compared to the literature as well as noisy responses are attributed to the high volume of our humidity measurement chamber, making it difficult to provide fast adjustment and uniform level of humidity inside the chamber. However, the differences between the performances are still distinguishable.


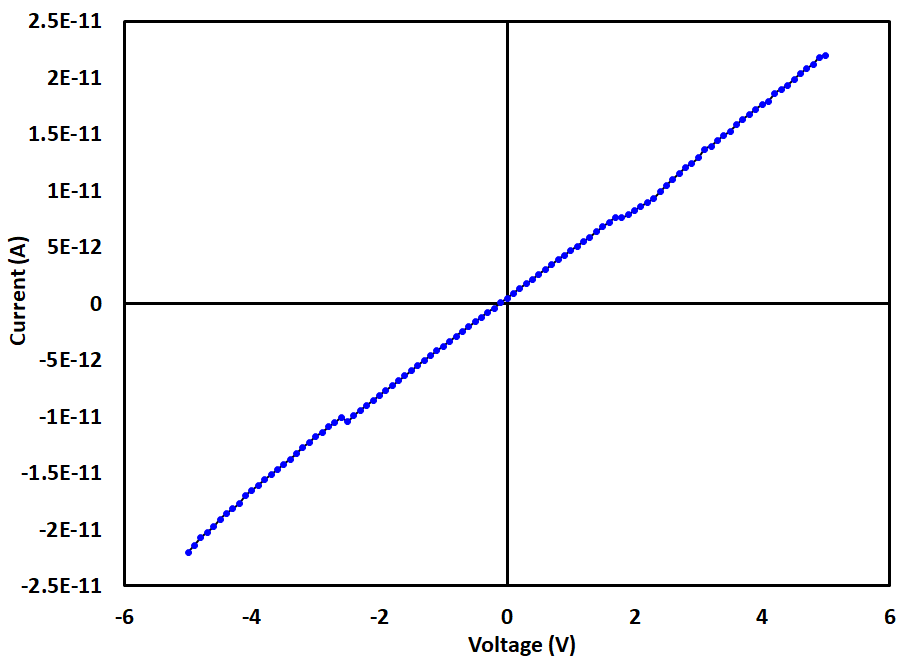


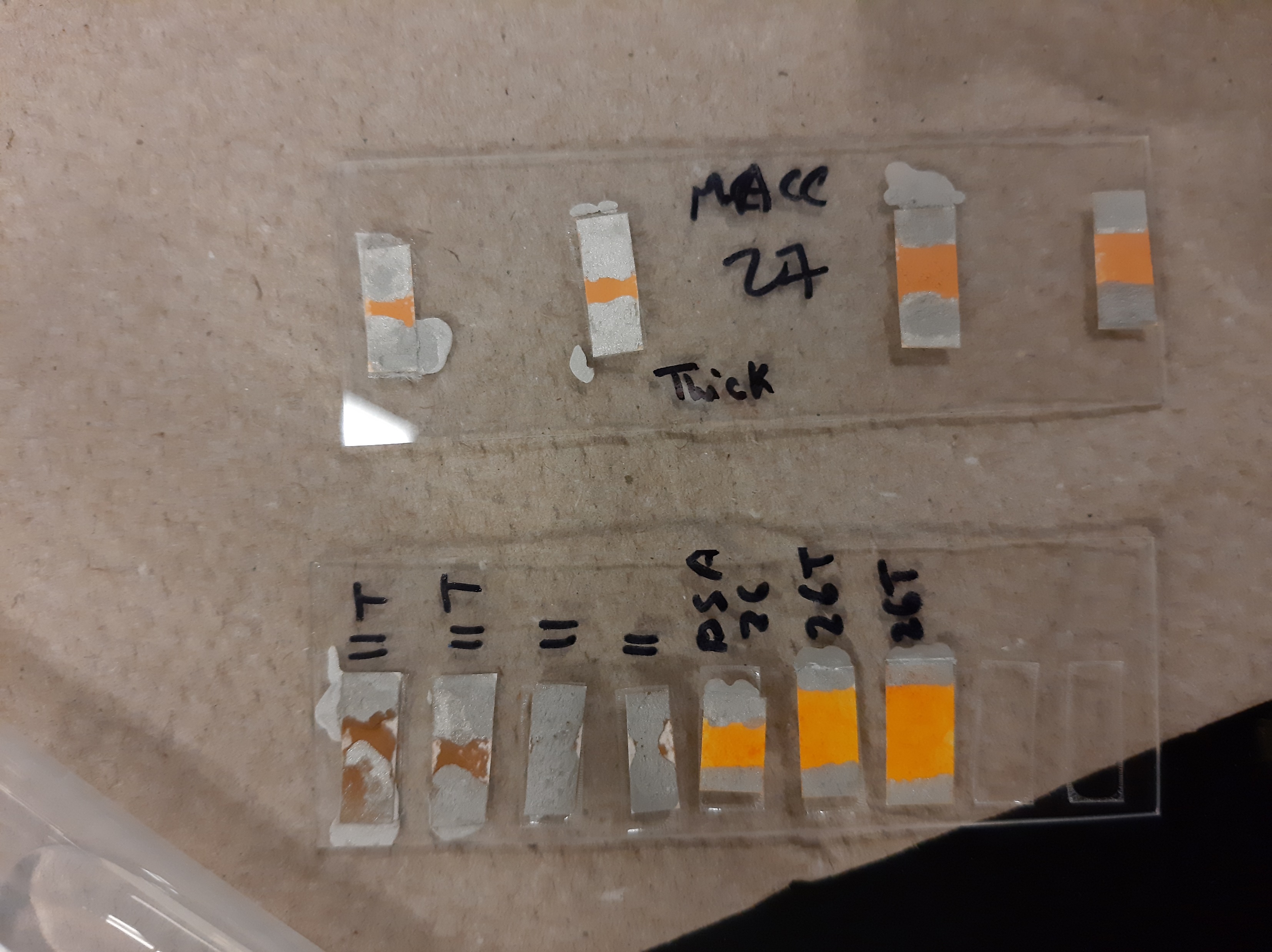


**Figure S17.** I–V characteristics of the fabricated paper based CH_3_NH_3_PbBr_3_ device in linear scale.


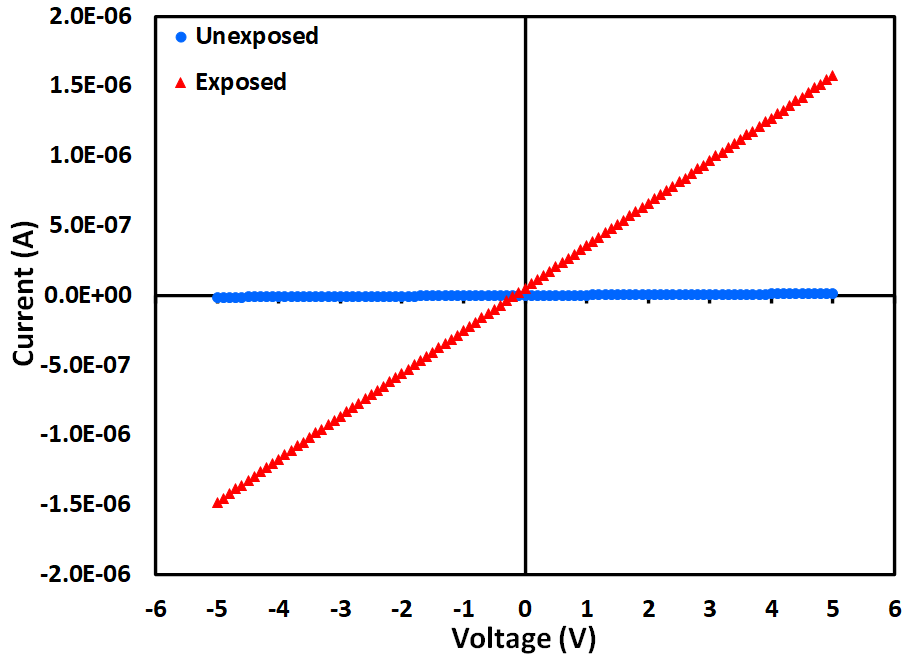


**Figure S18.** I–V characteristics of the fabricated paper based CH_3_NH_3_PbI_3_ device as a function of ammonia exposure in linear scale.


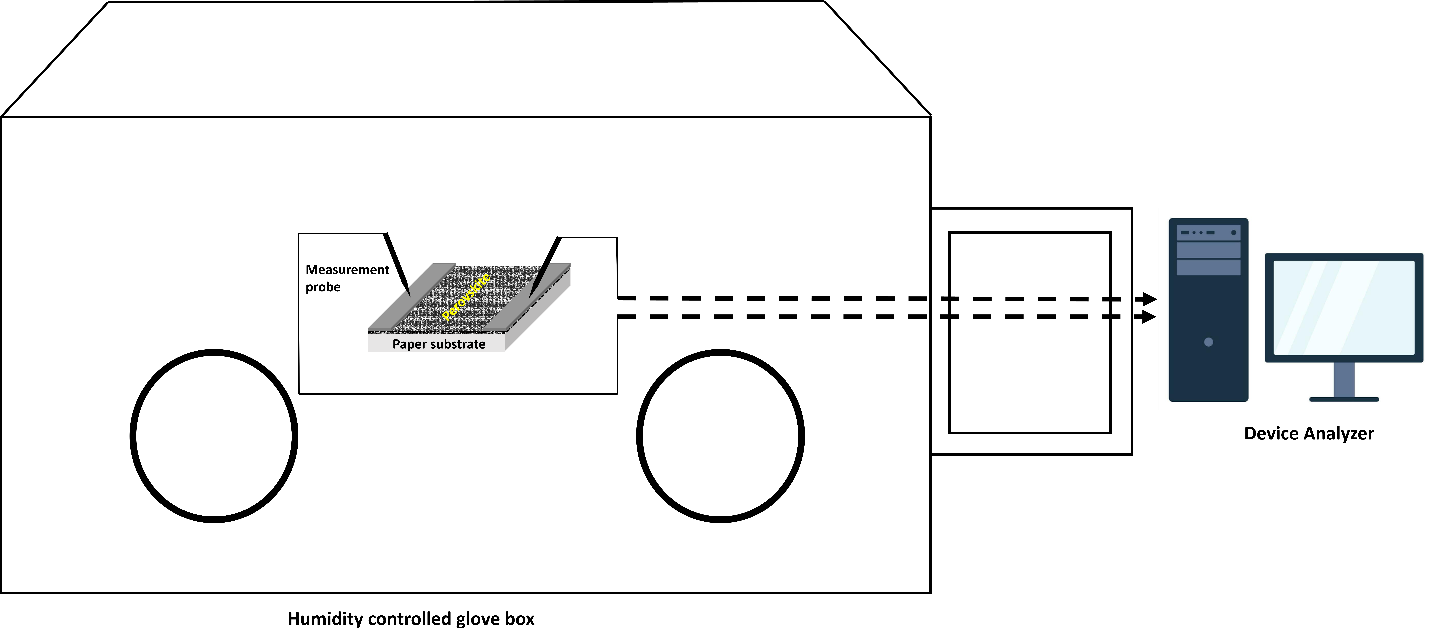


**Figure S19.** Device measurement configurations used in this study for the humidity sensing application.

**Table S1.** Summary of humidity sensors based on halide perovskites.

| Compound | Detection range (%) | Mode of detection | Reference |
| --- | --- | --- | --- |
| CH_3_NH_3_PbBr_3_ | 7-98 | Optical | ^1^ |
| Cs_2_InBr_5_⋅H_2_O | 30-80 | Optical | ^2^ |
| CsPbBr_3_ | 30-90 | Optical | ^3^ |
| Cs_4_PbX_6_ (X = Cl, Br, I) | 40-80 | Optical | ^4^ |
| CsPbBr_3_ | 30–95 | Optical | ^5^ |
| (CH_3_NH_3_)_2_CuBr_4_ | 7–98 | Optical | ^6^ |
| CH_3_NH_3_PbI_3−x_Cl_x_ | 30-90 | Electrical | ^7^ |
| CH_3_NH_3_PbI_3_ | ≤80 (linearity 40-80) | Electrical | ^8^ |
| CH_3_NH_3_PbI_3−x_Cl_x_ | 32-97 | Electrical | ^9^ |
| Cs_2_PdBr_6_ | 11-95 (linearity 33-95) | Electrical | ^10^ |
| Cs_2_TeCl_6_ | 5-90 | Electrical | ^11^ |
| CH_3_NH_3_PbI_3_ | 10-95 | Electrical | ^12^ |
| Cs_2_BiAgBr_6_ | 5-75 (linearity 15-78) | Electrical | ^13^ |
| CsPb_2_Br_5_/BaTiO_3_ | 25-95 | Electrical | ^14^ |
| CsPbBr_3_ | 11–95 (linearity 11-67) | Electrical | ^15^ |
| Cs_3_Cu_2_Br_5_ | 12-95 (linearity 33-95) | Electrical | ^16^ |
| Cs_3_Sb_2_Br_9_ | 2-95 | Electrical | ^17^ |
| Cs_2_PdBr_6_ | 11-85 | Electrical | ^18^ |
| Cs_2_lnCl_5_(H_2_O) | 11-97 | Electrical | ^19^ |
| K_2_CuBr_3_ | 12-95 (linearity 33-95) | Electrical | ^20^ |
| Cs_2_XCl_6_ (X = Hf, Zr, Te) | 11-85 | Electrical | ^21^ |
| K_2_CuBr_3_ | 11-95 | Electrical | ^22^ |
| Cs_2_TeI_6_ | 11-97 | Electrical | ^23^ |
| Cs_2_SnCl_6_ | 11-95 | Electrical | ^24^ |
| Cs_2_TeCl_6_ | 11-97 | Electrical | ^25^ |
| **CH_3_NH_3_PbI_3_** | **11-95** | **Electrical** | **This work** |

**References:**

1. W. Xu, F. M. Li, Z. X. Cai, Y. R. Wang, F. Luo and X. Chen, *J. Mater. Chem. C*, 2016, **4**, 9651-9655.

2. L. Zhou, J. F. Liao, Z. G. Huang, J. H. Wei, X. D. Wang, W. G. Li, H. Y. Chen, D. B. Kuang and C. Y. Su, *Angew. Chem.-Int. Edit.*, 2019, **58**, 5277-5281.

3. R. Li, J. Yu, S. Wang, Y. Shi, Z. Wang, K. Wang, Z. Ni, X. Yang, Z. Wei and R. Chen, *Nanoscale*, 2020, DOI: 10.1039/d0nr01889a.

4. X. Y. Yu, L. Z. Wu, H. C. Hu, M. Chen, Y. S. Tan, D. Yang, Q. Pan, Q. X. Zhong, T. Supasai and Q. Zhang, *Langmuir*, 2018, **34**, 10363-10370.

5. K. Sandeep, Varanasi, INDIA, 2020.

6. H. Lee, D. Lee, H. Jin, D. Baek, M. K. Kim, J. Cha, S. K. Kim and M. Kim, *Nanoscale Adv.*, 2022, **4**.

7. K. K. Ren, L. Huang, S. H. Yue, S. D. Lu, K. Liu, M. Azam, Z. J. Wang, Z. M. Wei, S. C. Qu and Z. G. Wang, *J. Mater. Chem. C*, 2017, **5**, 2504-2508.

8. A. S. Ilin, P. A. Forsh, M. N. Martyshov, A. G. Kazanskii, E. A. Forsh and P. K. Kashkarov, *ChemistrySelect*, 2020, **5**, 6705-6708.

9. L. Hu, G. Shao, T. Jiang, D. B. Li, X. L. Lv, H. Y. Wang, X. S. Liu, H. S. Song, J. Tang and H. Liu, *ACS Appl. Mater. Interfaces*, 2015, **7**, 25113-25120.

10. W. Ye, Q. Cao, X. F. Cheng, C. Yu, J. H. He and J. M. Lu, *Journal of Materials Chemistry A*, 2020, **8**, 17675-17682.

11. C. J. Pi, X. Yu, W. Q. Chen, L. L. Yang, C. Wang, Z. C. Liu, Y. Y. Wang, J. B. Qiu, B. T. Liu and X. H. Xu, *Materials Advances*, 2021, **2**, 1043-1049.

12. M. A. Haque, A. Syed, F. H. Akhtar, R. Sheyate, S. Singh, K. V. Peinemann, D. Baran and T. Wu, *ACS Appl. Mater. Interfaces*, 2019, **11**, 29821-29829.

13. Z. H. Weng, J. J. Qin, A. A. Umar, J. Wang, X. Zhang, H. L. Wang, X. L. Cui, X. G. Li, L. R. Zheng and Y. Q. Zhan, *Advanced Functional Materials*, 2019, **29**, 9.

14. M. Y. Cho, S. Kim, I. S. Kim, E. S. Kim, Z. J. Wang, N. Y. Kim, S. W. Kim and J. M. Oh, *Advanced Functional Materials*, 2020, **30**, 12.

15. Z. L. Wu, J. Yang, X. Sun, Y. J. Wu, L. Wang, G. Meng, D. L. Kuang, X. Z. Guo, W. J. Qu, B. S. Du, C. Y. Liang, X. D. Fang, X. S. Tang and Y. He, *Sensors and Actuators B-Chemical*, 2021, **337**.

16. Y. Y. Huang, C. Y. Liang, D. F. Wu, Q. J. Chang, L. B. Liu, H. B. Liu, X. S. Tang, Y. He and J. Qiu, *J. Phys. Chem. Lett.*, 2021, **12**, 3401-3409.

17. H. You, D. F. Wu, J. Wang, J. He, X. Y. Kuang, C. L. Li, F. W. Guo, D. K. Zhang, Q. Qi and X. S. Tang, *Appl. Phys. Lett.*, 2023, **122**.

18. L. S. Zhao, P. D. Ouyang, X. Y. Yi and G. Q. Li, *Applied Surface Science*, 2024, **649**.

19. Y. F. Liu, C. T. Li, L. X. Zhang, M. X. Chong and L. J. Bie, *Scripta Materialia*, 2023, **228**.

20. C. Y. Liang, Y. Y. Huang, Y. J. Shi, D. F. Wu, Q. J. Chang, B. S. Du, X. Z. Guo, D. L. Kuang, Z. L. Wu, C. J. Zhao, X. S. Tang, J. Qiu and Y. He, *Acs Applied Electronic Materials*, 2022, **4**, 1592-1602.

21. Q. J. Chang, D. F. Wu, Y. Y. Huang, C. Y. Liang, L. B. Liu, H. B. Liu, Y. Liu, J. Qiu, X. S. Tang and G. Q. Han, *Applied Surface Science*, 2022, **603**.

22. Q. J. Chang, D. F. Wu, Y. Y. Huang, C. Y. Liang, L. B. Liu, H. B. Liu, Y. He, Q. Huang, J. Qiu and X. S. Tang, *Sensors and Actuators B-Chemical*, 2022, **367**.

23. M. M. Zhao, C. T. Li, L. X. Zhang, L. J. Bie and Y. Y. Yin, *Materials Letters*, 2023, **334**.

24. M. Y. Pi, D. F. Wu, J. Wang, K. Chen, J. He, J. Yang, D. K. Zhang, S. J. Chen and X. S. Tang, *Sensors and Actuators B-Chemical*, 2022, **354**.

25. M. X. Chong, C. T. Li, L. X. Zhang and L. J. Bie, *Sens. Actuator A-Phys.*, 2023, **351**.
